# Emerging therapeutic potential of anti-psychotic drugs in the management of human glioma: A comprehensive review

**DOI:** 10.18632/oncotarget.26994

**Published:** 2019-06-11

**Authors:** Muhamad N.A. Kamarudin, Ishwar Parhar

**Affiliations:** ^1^ Brain Research Institute Monash Sunway (BRIMS), Jeffrey Cheah School of Medicine and Health Sciences, Monash University Malaysia, Selangor, Malaysia

**Keywords:** glioma, anti-psychotic drugs, typical anti-psychotic, atypical anti-psychotic, valproic acid

## Abstract

Despite numerous advancements in the last decade, human gliomas such as astrocytoma and glioblastoma multiforme have the worst prognoses among all cancers. Anti-psychotic drugs are commonly prescribed to treat mental disorders among cancer patients, and growing empirical evidence has revealed their antitumor, anti-metastatic, anti-angiogenic, anti-proliferative, chemo-preventive, and neo-adjuvant efficacies in various *in vitro*, *in vivo*, and clinical glioma models. Anti-psychotic drugs have drawn the attention of physicians and researchers owing to their beneficial effects in the prevention and treatment of gliomas. This review highlights data on the therapeutic potential of various anti-psychotic drugs as anti-proliferative, chemopreventive, and anti-angiogenic agents in various glioma models via the modulation of upstream and downstream molecular targets involved in apoptosis, autophagy, oxidative stress, inflammation, and the cell cycle in *in vitro* and *in vivo* preclinical and clinical stages among glioma patients. The ability of anti-psychotic drugs to modulate various signaling pathways and multidrug resistance-conferring proteins that enhance the efficacy of chemotherapeutic drugs with low side-effects exemplifies their great potential as neo-adjuvants and potential chemotherapeutics in single or multimodal treatment approach. Moreover, anti-psychotic drugs confer the ability to induce glioma into oligodendrocyte-like cells and neuronal-like phenotype cells with reversal of epigenetic alterations through inhibition of histone deacetylase further rationalize their use in glioma treatment. The improved understanding of anti-psychotic drugs as potential chemotherapeutic drugs or as neo-adjuvants will provide better information for their use globally as affordable, well-tolerated, and effective anticancer agents for human glioma.

## INTRODUCTION

Cancer is prevalent globally, and although with growing health apprehension and therapeutic developments, its mortality rate is still alarming [1, 2]. Moreover, a recent report in 2017 estimated 23,800 new cases of brain and central nervous tumor (CNS) tumors out of 1,688,780 new cases estimated in the United States alone. Despite the low prevalence as compared with other cancers, the statistical rate of mortality from glioma and other related brain tumors is estimated to be around 70% in the year 2017 alone [3]. Glioma, which refers to tumors of glial cell origin, is the most common type of central CNS tumor and constitutes more than 30% of all primary brain and CNS malignant tumors [4]. According to the World Health Organization, glioma is classified into four different classes with different grades: astrocytoma (grade I–II), anaplastic astrocytoma (grade III), oligodendrogliomas, ependymomas, mixed gliomas, and glioblastoma multiforme (GBM) (Grade IV). Among them, human GBM is known as the most lethal form of glioma with the worst prognosis. Currently, there is no available cure for GBM despite some therapeutic advancements in the last decade. The common multimodal treatment for GBM, known as Stupp’s regimen, consists of surgical resection which is followed by six weeks of radiation and concurrent daily intake of the chemotherapeutic drug temozolomide (3-methyl-4-oxoimidazo[5,1-d] [1, 2, 3, 5] tetrazine-8-carboxamide, Tmz) (with treatment lasting at least 6 months) [5, 6]. Tmz is an alkylating agent that is rapidly converted at physiological pH to a short-lived active compound, 5-(3-methyltriazen-1-yl) imidazole-4-carboxamide (MTIC), and further hydrolyzed to 5-amino-imidazole-4-carboxamide (AIC) and methylhydrazine [5]. The methylation of N-7 and O-6 sites on guanine and the O-3 site on adenine residues confers Tmz cytotoxicity leading to cell cycle arrest at G_2_/M and cell death. This treatment regimen remains as the primary standard care for GBM patients for the past ten years, and typically results in a median overall patient survival of 14.6 months from date of surgical diagnosis [7, 8].

The complexity of tumor coupled with high chemoresistance and chemotoxicity further dampen the efficacy of chemotherapy drugs leading to cancer recurrence with poor therapeutic indexes [9, 10]. Moreover, current chemotherapeutic agents are incapable of exclusively targeting tumor cells and thus causing adverse side-effects such as anemia, bleeding, diarrhea, hair loss, nausea, vomiting and immunosuppression that increases the chance of infection [11, 12]. Glioma cells can develop resistance against Tmz by inducing the repair of DNA damage via expression of proteins such as O6-alkylguanine DNA alkyltransferase (AGT) that demethylates Tmz-methylated guanosine encoded in humans by the O-6-methylguanine-DNA methyltransferase (MGMT) gene [13]. Other than chemotherapy, human GBM is also treated by immunotherapy approaches such as monoclonal antibodies (bevacizumab, nivolumab), a peptide vaccine (rindopepimut), checkpoint inhibitors, dendritic vaccines, and adopted T cells (chimeric antigen receptors (CARs)) that aim to provide a more specific and defined immunization strategy in mediating tumor cell killing. The addition of Novo-TTF (tumor-treating fields) to the Stupp’s regimen resulted in increased overall survival (OS; 19.6 months) and progression-free survival (PFS; 7.1 months) compared with patients who received a conventional Stupp’s regimen [14]. Although this improved Stupp’s regimen was suggested to be the new standard of care against GBM, the treatment only increased patients’ OS to approximately 19 months without significant improvement in prognoses.

**Figure 1:**
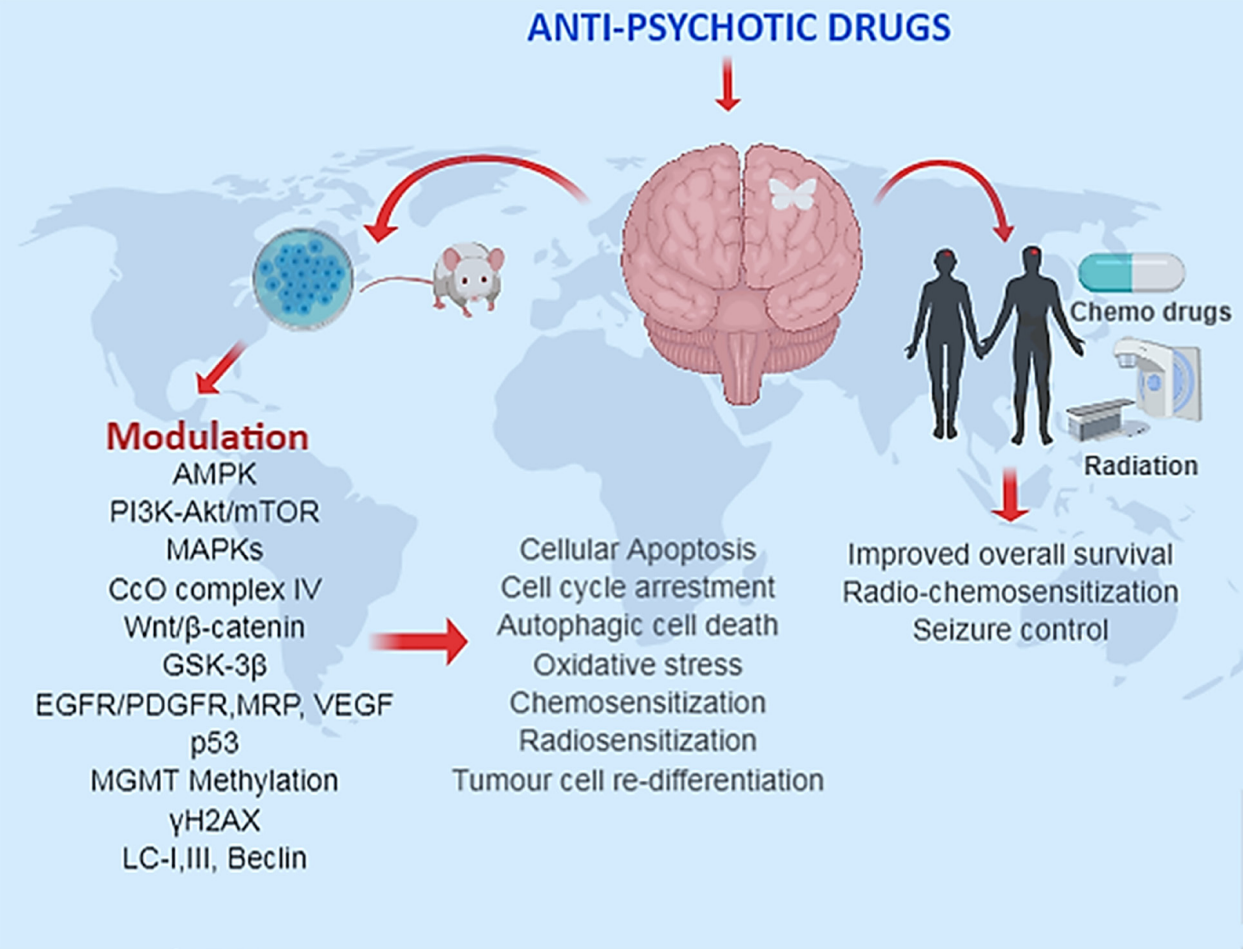
The summarized use of anti-psychotic drugs in preclinical and clinical glioma studies.

**Figure 2:**
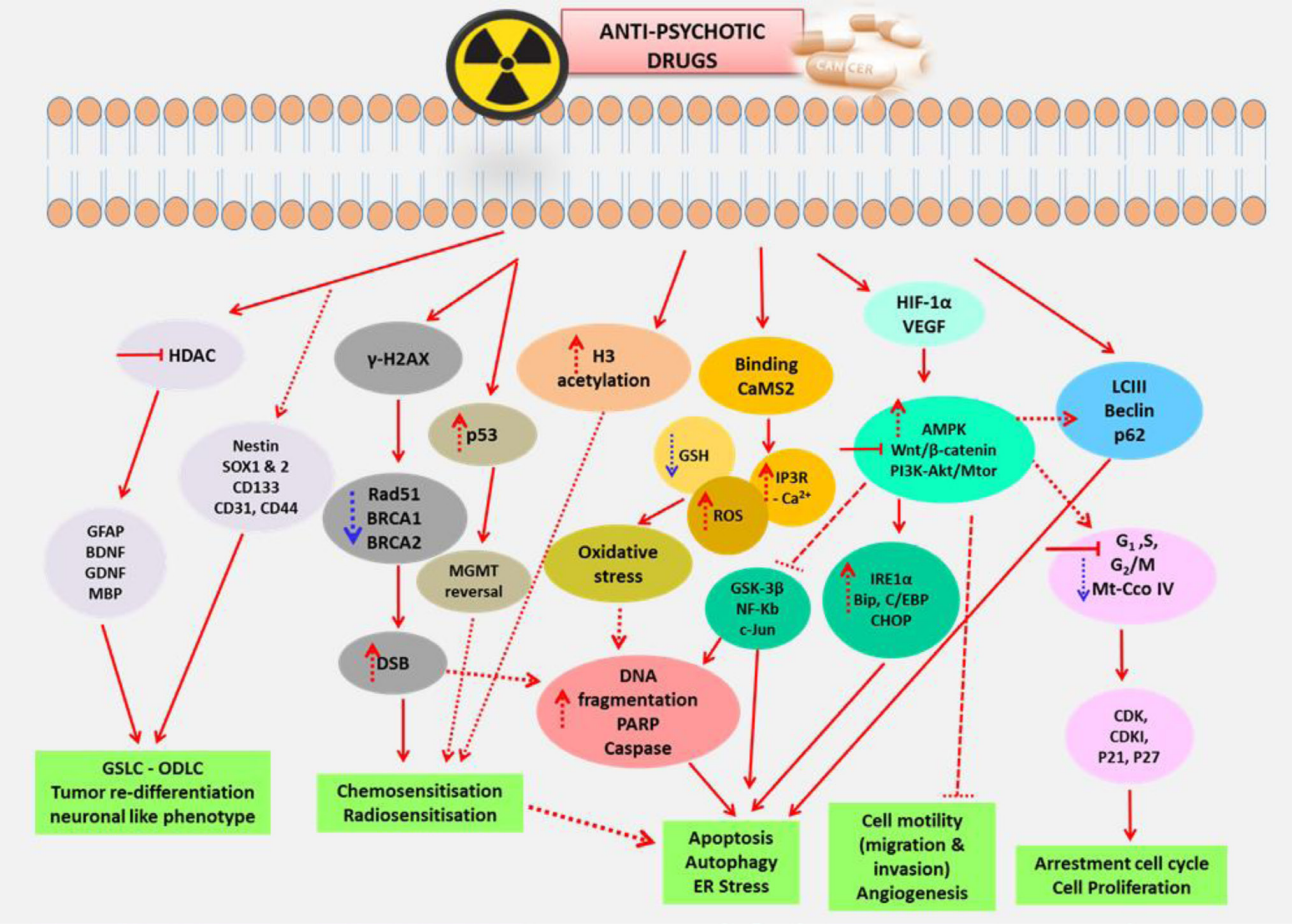
The summarized mechanistic pathways and molecular targets of anti-psychotic drugs. Various mechanisms and molecular targets induced by anti-psychotic drugs which includes inactivation of AMPK, PI3K-Akt/mTOR, Wnt/β-catenin, inhibition of HDAC and modulation of GSCs and NSCs markers, γ-H2Ax, p53 hyperacetylation, histone acetylation and induction of oxidative stress that result in their multimodal therapeutic effects.

Although usually administered as the front line of treatment for mental disorders, anti-psychotic drugs are increasingly prescribed among cancer patients to improve their quality of life [15, 16]. Generally, anti-psychotic drugs are grouped into two main classes; the typical and atypical anti-psychotic drugs. A majority of the anti-psychotic drugs are derived from the tricyclic phenothiazine chlorpromazine that was originally prescribed for neuropsychiatric disorders. Most of the first-generation of anti-psychotic drugs were designed to weaken abnormal dopaminergic function by targeting dopamine D2 receptors which can induce Parkinson-like symptoms [17, 18]. Owing to this fact, second generation or atypical anti-psychotic drugs were developed (derived from clozapine) that function by antagonizing the serotonin 2A receptor (5-HT2A) [19, 20]. Following this, advancement in the pharmaceutical industry steered the modification of chlorpromazine leading to the development of tricyclic anti-depressants and selective serotonin reuptake inhibitors. Generally, cancer patients develop psychological issues such as feelings of despair and anxiety which further lead to depression following diagnosis (Fitzgerald *et al*., 2015; Walker *et al*., 2013). Moreover, the various side-effects from chemotherapy and radiation can further exacerbate psychological symptoms. Therefore, anti-psychotic drugs are prescribed as a method to integrate mental healthcare during cancer treatment, which interestingly demonstrated a lowering of the cancer incidence and grade as reported in the second SMaRT (Symptom Management Research Trial in Oncology-2) study [21]. This observation is further strengthened by the numerous preclinical and clinical reports that demonstrate the anti-glioma efficacy of anti-psychotic drugs as monotherapy agents and adjuncts in polytherapy treatment settings (Figure 1). This review manuscript covers the initial until the latest preclinical (Supplementary Table 1) and clinical with specific case findings of major classes and types of anti-psychotic drugs. Additionally, the current review also highlights the contradictory findings, further in-depth molecular mechanisms and future perspectives with critical review on repurposing anti-psychotic drugs as potential therapeutic for glioma (Figure 2). Therefore, this review aims to provide comprehensive and up-to-date preclinical and clinical reports of anti-psychotic drug use as anti-glioma agents that would further rebrand and justify their repurposed use in human glioma management.

## THE PRECLINICAL EXPERIMENTAL EVIDENCE OF THE USE OF ANTI-PSYCHOTIC DRUGS IN TREATING GLIOMA

### Typical anti-psychotic drugs as potential modality in glioma

Haloperidol, an antagonist of sigma-1 and sigma-2 receptors, was first reported to reduce the global growth ratios and cell division number ratios in both U373 and T98G cells when treated with 5 μM for 48 and 72 h [22]. In a more recent finding, (R)-(+)-MRJF4 and (S)-(-)-MRJF4, asymmetric of (±)-MRJF4, a novel ester prodrug of haloperidol metabolite II (HP-mII) were shown to be more lipophilic towards the blood-brain barrier and induced apoptotic cell death in rat c6 glioma in a concentration- and time-dependent manner (0 to 5 μM, 14–72 h) with IC_50_ values of 5 μM [23]. Additionally, both compounds were shown to induce cell cycle arrestment at S-phase (72 h), suppressed C6 cell migration and markedly increased histone3 (H3) acetylation.

In one of the early investigations, a group of phenothiazine drugs (thioridazine, perphenazine, chlorpromazine, and fluphenazine) concentration-dependently reduced viability (up to 90% reduction as compared to control) of C6 cells following treatment exposure for 24 h, with IC_50_ values of 13.7 μM (thioridazine), 15.8 μM (perphenazine), 18.8 μM (chlorpromazine) and 19 μM (fluphenazine) [24]. In addition, exposure to fluphenazine alone (1–100 μM) resulted in greater cytotoxicity when compared with its N-mustard analog (irreversible calmodulin antagonist), and in combination with SKF10047 (prototype σ1 receptor ligand). Further evaluation revealed that 24 h exposure to thioridazine, fluphenazine, and perphenazine (6–50 μM) concentration-dependently increased DNA fragmentation (up to 94%). Thioridazine (10–100 μM) was further tested against normal primary mouse cells and selected neurons where it demonstrated lower selectivity as compared with higher sensitivity in glioma and neuroblastoma cells. Moreover, thioridazine (12.5 μM) induced apoptotic cell death as evidenced by Hoechst 33342/PI staining while it (25 and 50 μM, 4 h) significantly elevated caspase-3 activity up to 30-fold in SH-SY5Y cells. Recently, it was demonstrated that a single treatment with perphenazine and prochlorperazine induced concentration-dependent cell viability, with EC_50_ values of 0.98 μM and 0.97 μM, respectively [25]. In a previous study, exposure to irradiation and temozolomide in combination with perphenazine (0–10 μM) for 7 days resulted in a concentration-dependent reduction of U87 cell viability with an LD_50_ of 4 Gy (irradiation) and an LC_50_ of 6.8 μM (perphenazine) [26]. Furthermore, when cells were treated with Tmz (5, 10 or 15 μM, for 7 days) in combination with perphenazine (5 μM), an additive effect in the reduction of cell content was noticed, whereas a synergistic effect of cell content reduction was observed with the combination treatment using imatinib (10 μM). However, the synergistic effect of imatinib and perphenazine was not related to cell cycle arrest, decreased ATP production or phosphorylation status of Akt and MAPK proteins. In a different study, treatment with both thioridazine and fluphenazine (1– 10 μM, 72 h) significantly reduced the GBM8401 and U87MG cell viability as compared with Tmz at the same range of concentrations [27]. Additionally, thioridazine concentration-dependently attenuated GBM8401 colonic formation while activating autophagic cell death through the upregulation of LC3-II, Beclin1, and cleavage of caspase-8 and -3, which subsequently activated PARP via the activation of AMPK protein and inactivation of the PI3K-Akt pathway through the inhibition of RAPTOR. Furthermore, thioridazine was shown to induce cell death through the endoplasmic reticulum (ER) stress pathway, as demonstrated by the increased accumulation of inositol-requiring enzyme 1 alpha (IRE1α)) and the binding of immunoglobulin Bip protein and C/EBP homologous protein (CHOP)). When tested in U87-xenograft nude mice, treatment with thioridazine (5 mg/kg/day, 5 days/week) significantly activated autophagic-induced suppression of tumorigenesis, which was evidenced by the increased expression of LC3-II and a concomitant reduction in tumor size. Oncolytic viral therapy has been viewed as a potential therapeutic tool to activate the immune system for killing cancer cells without harming healthy cells. Fluphenazine (0–10 μM, 24 h) was reported to exert its function by acting on dopamine receptors to sensitize the apoptotic (prolonged caspase-3/7 activation) and necrotic (enhancement of LDH) potential of OV Delta24-RGD in glioblastoma stem-like cells (GSCs) *in vitro* [28].

Another type of phenothiazine, trifluoperazine, was reported to induce both concentration-dependent (1, 2, 5, 10, and 20 mmol/L) and time-dependent (24–72 h) reductions in viability of U87MG glioblastoma cells. When used above a concentration of 2 mmol/L, trifluoperazine inhibited the anchorage-independent growth, motility, and invasion with a half-maximal effective concentration of approximately 10 mmol/L) [29]. Moreover, treatment with trifluoperazine led to its binding with calmodulin subtype 2 (CaMS2), which led to CAMS2 dissociation from IP3R leading to the opening of IP3R subtype 1 and 2 and concomitantly elevated the release of Ca^2+^ ions. In an animal study, treatment with trifluoperazine (5 mg/kg/day) was shown to inhibit the growth of tumors in U87MG-xenograft nude mice at day 21 with a 50% reduction in tumor weight, although such treatment did not increase overall survival time. Following this study, fourteen trifluoperazine analogs were synthesized and tested in U87MG and GBL28 human glioblastoma patient-derived primary cells [30]. The MTT test further revealed that treatment with two analogs (1–20 μM for 24 h), 10-(4-(4-(Pyrrolidin-1-yl)piperidin-1-yl)butyl)-2-(trifluoromethyl)-10H-phenothiazine (3dc) and 10-(4-([1,40-Bipiperidin]-10-yl)butyl)-2-(trifluoromethyl)-10H-phenothiazine (3dd) exhibited higher cytotoxicity (4-5 times) than trifluoperazine, with IC_50_ values of 2.3 and 2.2 μM, respectively in U87MG cells and IC_50_ of 2.2 and 2.1 μM, respectively in GBL28 primary cells. The authors described that although both analogs exhibited some toxicity in normal NSC neural cells, they demonstrated reasonable selectivity with significant higher cytotoxicity against GBM cells. Moreover, molecular modeling suggested that the analogs promoted the release of intracellular Ca^2+^ ions which led to glioma cell death. More importantly, when tested against xenograft U87MG nude mice, analog 3dc was found to significantly decrease brain tumor size (by 88%), with subsequent prolonged survival time (increased by 6 days). In a different report, trifluoperazine treatment was shown to block GBM cell survival by inhibiting autophagy that reduced resistance against radio-sensitivity in GBM models [31]. Exposure to trifluoperazine (0–30 μM, 48 h) concentration-dependently decreased the U251, U87 and P3 (a primary human biopsy) cell viability with IC_50_ values of 16, 15, and 15.5 μM, respectively. Trifluoperazine treatment (0–10 μM, 24–48 h) significantly decreased the total 5-ethynyl-2’-deoxyuridine (EdU)-positive cells, clonogenic formation, and markedly elevated the increased caspase-3/7. Although the author reported significant selectivity of trifluoperazine in GBM cells (*P* < 0.05), nevertheless, the small range different value of IC_50_ between GBM and NHA cells (IC_50_ 22.5 μM) sparks an interesting query regarding the efficacy versus toxicity of trifluoperazine usage since IC_50_ values of TFP in all GBM cells demonstrated significant cytotoxicity in NHA cells. Nevertheless, the authors demonstrated that TFP (10 μM, 48 h) disrupted the acidification of lysosomes by up-regulating LC3B-II and p62 expression similar to the positive control, bafilomycin A1 (BAF, 100 nM for 48 h). Furthermore, subsequent trifluoperazine (5 μM) addition for 24 h significantly enhanced radiation (4 Gy)-induced double-strand breaks (DSBs) by prolonging the γ-H2AX signal (~24 h post-irradiation) and downregulating the Rad51 and the associated DNA repair proteins BRCA1 and BRCA2 in U251 and U87 cells (27% and 21.6%, respectively) when compared with radiation alone (signal decreased after 6 h of radiation). This radio-sensitization effect produced by trifluoperazine was suggested to be mediated by its ability to suppress the cathepsin B and particularly, cathepsin L that also justified the inhibition of autophagy. In xenograft orthotopic nude mice U251 and P3 models, trifluoperazine (1 mg/kg, 5 days/week) in combination with radiation (5 Gy) significantly decreased the Ki67 proliferation index which led to improvement in the median survival time to 46 days, as compared with the 29.7 days with radiation alone. Moreover, the combination treatment paradigm also markedly decreased Rad51-positive cells, with a significant elevation of γ-H2AX as compared with radiation alone, which led the authors to suggest trifluoperazine as a novel autophagy inhibitor with radio-sensitization capability in GBM models.

An early study in 1994 first demonstrated that chlorpromazine (10 mg/kg body weight, on day 4 of inoculation) in combination with 1,3-bis(2-chloroethyl-l)-nitrosourea (BCNU) (10 mg/kg body weight, on day 3 of inoculation) exhibited significant tumor growth suppression in rats injected with RG2 glioma cells [32]. However, neither chlorpromazine nor BCNU treatment alone provided significant tumor growth inhibition, which exemplifies the synergism between chlorpromazine and BCNU in suppressing glioma growth. Almost two decades later, chlorpromazine was reported to suppress U87MG cell proliferation and long-term clonogenic survival by promoting autophagic cell death and increased accumulation of the microtubule-associated protein 1 LC3-II by mitigating the activation of the PI3K-Akt/mTOR pathway [33]. The same study demonstrated transient knockdown of Beclin 1, as well as exogenous expression of Akt protein partially blocked LC3-II formation that is essential in inducing autophagic cell death. The authors further reported that chlorpromazine treatment significantly induced the autophagy responsible for suppressing the tumor growth in xenograft U87MG nude mice. Recently, treatment with chlorpromazine was shown to be beneficial in mitigating cell proliferation in Tmz chemo-resistant cells and glioma stem cells by inhibiting cytochrome *c* oxidase (CcO) complex IV mitochondrial activity [34]. Chlorpromazine suppressed U251-derived TMZ-resistant (UTMZ) cells (24 h) with an IC_50_ of 13.12 ± 2.8 μM and blocked the anchorage-independent growth (10 μM) and concentration-dependently (2.5–10 μM) mitigated tumor neurosphere formation. Treatment with chlorpromazine (2 μM) displayed a concentration-dependent reduction of the CcO activity of complex IV and a non-competitive inhibition of cyt *c* with a decreased Vmax value of 50% (870 ± 57 to 375 ± 24 pmol/sec/mg) in UTMZ cells. Moreover, chlorpromazine concentration-dependently inhibited CcO activity (10 - 50 μM) in GSCs derived from both the J × 12 and J × 39 xenolines, which led to the significant reduction in the potential of stem cell frequency, and the frequency of self-renewing cells (2.5–5 μM) in both xenolines. In previous work, the authors demonstrated that acquisition of chemoresistance in glioma cells is influenced by the switching of CcO subunit 4 isoform 2 (COX4-2) expression to COX4-1. In a recent study, they showed that chlorpromazine arrested the cell cycle at the G_1_ phase (70.4% (10 μM) and 73.1% (20 μM)) in UTMZ cells expressing COX4-1.

Since the upregulation of CcO subunit 4 isoform 1 (COX4-1) coupled with elevated Cco activity can render glioma cells resistant towards Tmz, the authors suggested that the disruption of TMZ-resistance by chlorpromazine was primarily mediated through interference with metabolic machinery at the mitochondrial level. Furthermore, molecular modeling through computer-simulated docking studies revealed that chlorpromazine possesses higher binding capacity to CcO expressing COX4-1 than to CcO expressing COX4-2, which generates a steric hindrance responsible for the blockade of COX11 from interacting with the remainder of the CcO complex. When tested in the xenograft orthotopic UTMZ mice model, intraperitoneal treatment with chlorpromazine (5 or 7 mg/kg, three times a week for 2 weeks) prolonged the median overall survival of mice from 18.5 days (control) to 22.5 days (5 mg/kg) and 25.0 days (7 mg/kg).

### Atypical anti-psychotic drugs in glioma

Clozapine, a dibenzodiazepine derivative with a piperazinyl side-chain exerts its anti-psychotic properties by antagonizing 5-hydroxytryptamine 2A (5-HT2A) and dopamine D1 and D4 receptors. Clozapine is the first atypical anti-psychotic drug that was reported to inhibit the proliferation of U-87MG human glioblastoma cells through the interruption of voltage-gated calcium channels and calmodulin (CaM) via the inactivation of Akt protein [35]. Exposure to 20 μM clozapine significantly suppressed the full activation of Akt protein (at Ser 473) as early as 15 min after exposure, and this inhibition was prolonged up to 4 h which concomitantly increased dephosphorylation of GSK-3β (Ser 9) and elevated its kinase activity. Additionally, clozapine (10 and 20 μM) also inhibited the ability of EGF to induce the full activation of Akt. Following these observations, clozapine treatment alone downregulated cyclin D1 expression which was reversed following the addition of lithium chloride (LiCl) and elevation of Ca^2+^ ions. The inhibition of cyclin D1 by clozapine alone was preceded by cell cycle arrest in the G_0_/G_1_ phase (increased population from 73.2% to 94.3%), which was greater than the inhibition by EGF or LY294002 alone (65% and 87.2%, respectively). However, pre-treatment of serum-starved U-87MG cells with clozapine (1 h) prior to the addition of EGF significantly reduced the population S and G2/M phases. Olanzapine, another atypical anti-psychotic drug that antagonizes the 5-HT2A and dopamine D2 receptor, enhanced the anti-proliferative activity of temozolomide while its treatment alone exhibited significant inhibition of proliferation in U87MG, A172, and two glioma stem-like cells with IC_50_ values ranging from 25 to 79.9 μM [36]. Olanzapine alone (10–40 μM) concentration-dependently reduced colony formation growth in U87MG cells while it suppressed migration with significant induction of cytostatic effects in A172 cells. The authors further demonstrated that olanzapine exposure induced early and late apoptotic events in U87MG cells, whereas it induced late apoptotic and necrotic cell death in A172 cells. Moreover, treatment with olanzapine alone was shown to reduce the phosphorylation of AMPK and suppressed WNT and c-Jun pathway activation by downregulating β-catenin and c-Jun levels.

The ability of atypical anti-psychotic drugs to modulate plasticity of GSCs was tested in different oxygen (hypoxic) glioma GSCs models that were isolated from T98G cells. In different hypoxic glioma models, treatment with mirtazapine (10 μM, 24 h) did not demonstrate any significant changes in cell viability of either glioma line in all oxygen models (hypoxia 1% oxygen, average hypoxia 2.5% oxygen, hypoxia-reoxygenation model 1% oxygen for 12 h followed by 3% oxygen for 12 h and standard laboratory conditions 20% oxygen) [37]. Nonetheless, mirtazapine was shown to increase normal human astrocyte (NHA) mitochondrial activity. In the average hypoxia model, mirtazapine treatment downregulated Sox1 and Sox2 expression to nearly 0% as compared with Tmz (4%, Sox1 and 1%, Sox2). In the 20% oxygen model, the expression of CD44, Sox1, and Sox2 were not detected, while Ki67 expression was decreased. These contradictory findings in each of the hypoxic models will be further discussed in the next section. Numerous studies have shown that neural stem cells (NSCs) that possess sufficient oncogenic mutation can adopt neoplastic transformation into GSLCs. Moreover, the attempt of re-differentiating GSLCs into astrocytes is further dampened by glial scar formation that hampers functional neural recovery. Nevertheless, oriented differentiation of GSLCs into oligodendrocytes seems to provide improved prognosis in glioma with the chance of influencing functional neural recovery. The ability of atypical anti-psychotic drugs in re-differentiating GSCs into ODLCs through suppression of GSK-3β phosphorylation and inactivation of the Wnt/β-catenin pathway is substantiated by treatment with quetiapine in GSCs purified from the glioblastoma cell line GL261 [38]. Increasing concentrations of quetiapine (0, 5, 10, 25, 50 and 100 μM) significantly decreased GSCs cell viability while exposure at 25 μM arrested the cell cycle at the G_2_/M phase. The same exposure of quetiapine in GSCs increased MBP-positive cells while increasing concentrations from 5–25 μM upregulated the ODLCs lineage marker MBP and Olig1 expression. Conversely, the number of GFAP-positive cells and GSCs marker SOX2 were reduced with the same treatment paradigms. Quetiapine treatment was also shown to significantly inhibit tumor growth as well as PCNA-positive cells in heterotopic GSC-xenografted nude mice and orthotropic xenografted C57 mice at day 21. When combined with Tmz, the suppression of tumor growth was synergistically and significantly enhanced.

Interestingly, quetiapine monotherapy was solely responsible for influencing the re-differentiation of GSCs into ODLCs *in vivo,* since its treatment alone, but not TMZ treatment, significantly downregulated the expression of GFAP, Sox2, Olig2, and vimentin, and upregulated MBP expression. In a recent research perspective article, the use of a multimodal combination of six repurposed marketed drugs (itraconazole, metformin, naproxen, pirfenidone, rifampin, and quetiapine) alongside Stepp’s regimen was suggested as a potential inhibitor of epithelial to mesenchymal transition (EMT), as cells that are post-EMT are known to acquire invasive properties with limited proliferative capability [39]. Moreover, EMT cells exhibit elevated expression of vimentin, TGF-β, β-catenin, and fibronectin, which make them resistant towards chemotherapy and actively motile. In addition, the formation of de-tyrosinated alpha-tubulin micro-tentacles during the transformation of EMT further assists glioblastoma cell migration and metastasis to distant sites. This suggested treatment regimen, known as EMT inhibiting sextet (EIS), aims to block six major target sites that confer glioblastoma invasive, metastatic, resistance, and proliferative properties (itraconazole as an inhibitor of Hedgehog signaling, metformin to inhibit AMP kinase (AMPK), naproxen to suppress Rac1, pirfenidone to impair the secretion of transforming growth factor-beta (TGF-β) and rifampin as a suppressor of Wnt signaling). Quetiapine is suggested as a blocker of the receptor activator NF-κB ligand (RANKL) since its signaling is thought to mediate the behaviors of EMT. Moreover, secretion of RANKL can augment glioblastoma invasive motility through paracrine signaling towards the non-malignant astrocytes in the nearby milieu. This in turn can induce astrocytes to produce TGF-β that boosts the process of glioma cell migration and invasion. Therefore, the use of quetiapine in EIS regimen is postulated to suppress the growth-enhancing cycle between glioma and nearby non-malignant astrocytes by virtue of its ability to impair RANKL and TGF-β secretion. Based on this research perspective, it is strongly suggested that the mooted EIS be undertaken and tested in a clinical trial as an attempt to target multiple resistance pathways that impair the current therapeutic efficacy of Stepp’s regimen and thus, improve prognosis. When compared with typical anti-psychotic drugs, it is rather fascinating and noteworthy that atypical anti-psychiatric drugs exert their anti-glioma functions mainly by modulating the plasticity of GSCs cells, which is extremely beneficial in mitigating GCSs-induced chemoresistance.

### Repositioning selective serotonin reuptake inhibitors (SSRIs) as glioma treatments

In an early report, treatment with fluoxetine (1 and 5 μM) was shown to induce apoptotic cell death in C6 cells as observed by the increased DNA fragmentation in the TUNEL assay [40]. Although no mechanistic data were reported, this observation was supported in a later study on C6 and SH-SY5Y cells [41]. Treatment with fluoxetine and paroxetine (0–50 μM for 24 h) led to a concentration-dependent reduction in cell viability that was followed by a concentration-dependent increase in DNA fragmentation and apoptotic morphological changes (treatment with 12 μM) in both cell lines. Paroxetine exhibited a more potent pro-apoptotic activity than fluoxetine, where treatment at 12 μM inhibited 80% of the cell viability as compared with 50 μM by fluoxetine. Moreover, paroxetine exposure at 50 μM decreased cell viability, with IC_50_ values of 71.6 μM and 53.8 μM in primary whole brain and neuronal cultures, respectively, which exemplified their selectivity towards C6 and SH-SY5Y cells (4-fold higher). Additionally, paroxetine (15 and 20 μM) induced apoptotic cell death as shown by increased caspase-3 activity that was also suppressed by the pre-addition of the caspase-3 inhibitor Ac-DEVD-CHO. Furthermore, the activation of caspase-3 by paroxetine treatment at 6 and 12 μM was preceded by the rapid and transient activation of phospho-c-Jun levels and subsequent mitochondrial release of Cytochrome *c* (Cyt. *c*) in C6 cells.

Similar to perphenazine, Tzadok [25] reported that a combination of the SSRIs sertraline (0–10 μM) and fluoxetine (0–20 μM) with increasing doses of irradiation (0–8 Gy) for 7 days also exhibited concentration-dependent reduction of U87 cell viability. They reported that the LC_50_ for the SSRIs combination was slightly higher than perphenazine treatment (sertraline, 8 μM and fluoxetine, 19 μM, respectively). Even though the combination of sertraline (7.5 μM) with increasing doses of Tmz (5–15 μM) and imatinib (10 μM) for 7 days resulted in additive and synergistic reduction of U87 cell content, treatment with irradiation (4 Gy) demonstrated less additive effect as observed in the cells. Moreover, combination treatment did not significantly alter or induce cell cycle arrest, although fluoxetine (15 μM), in combination with imatinib (10 μM), elevated the DNA fragmentation up to 43-fold without promoting caspase-3 activity. Nevertheless, imatinib and sertraline combination resulted in significant inhibition of pAkt (to 70%) whereas combination with fluoxetine suppressed pAkt (to 63 %) while increasing pMAPK expression (to 197 %). However, this study requires further in-depth mechanistic and animal studies to further elucidate the proper cell death mechanisms of these SSRIs. Comparative drug screening in GBM is a method that allows researchers to investigate and estimate the anticancer drug-drug interaction variability in whether they could exert their efficacy in a specific context or across a broader range of cases in glioma cells. Moreover, such methods allow the integration of different and wide-ranging molecular marker profiles, which in turn would enable the determination of novel biomarkers or target sites in glioma. Therefore, a group of researchers conducted comparative drug-drug interaction studies involving 31 marketed drugs that formed a matrix of 465 unique pairs, and calculated the interaction score in five different glioma cell lines, U87MG, U343MG, U373MG, A172, and T98G [42]. They reported that a synergistic interaction score when all of the cell lines were tested with a combination of rimcazole (10 μM) and sertraline (10 μM) for 48 h. Using isobolic analysis, the additive effects were seen with a combination treatment of rimcazole and sertraline, while synergistic actions between pterostilbene (20 μM) and sertraline (10 μM) resulted in a negative α-value and low combination indices. From these observations, the authors suggested the possibility of repurposing SSRI drugs in combination with the sigma receptor antagonist rimcazole and the antioxidant pterostilbene.

Fluoxetine has a therapeutic dose range of 20-60 mg/day, and its concentration in the brain can reach up to ~ 30 μM when used among depression patients. Fluoxetine treatment (at 25–30 μM for 24 h) was shown to decrease cell viability and induce apoptotic cell death in C6, U87, GBM8401 and Hs683 cells by promoting transmembrane extracellular Ca^2+^ influx via its direct binding to the to α-amino-3-hydroxy-5-methyl-4-isoxazolepropionic acid (AMPAR) receptor [43]. The role of fluoxetine in inducing mitochondrial-mediated apoptotic cell death was corroborated by the reduction of mitochondrial membrane potential (MMP) and leakage of cyt. *c* that concomitantly activated caspase-9, -3 and poly (ADP-ribose) polymerase (PARP) in U87 and GBM8401 cells, which was inhibited by the pan-caspase inhibitor, zVAD. Furthermore, oral administration of fluoxetine (10 mg/kg/day) in U87-xenograft nude mice reduced tumor growth at day 6, which became undetectable by day 12, and specifically increased the expression of caspase-3 in the brain tumor region, which was similar to Tmz treatment (5 mg/kg/day). Similar to the observation with mirtazapine (section 3.12), exposure to fluoxetine (10 μM, 24 h), escitalopram (10 μM, 24 h), and agomelatine (a melatonin receptor antagonist; 10 μM, 24 h) did not exert cytotoxicity in glioma lines in all oxygen models (hypoxia 1% oxygen, average hypoxia 2.5% oxygen, hypoxia-reoxygenation model 1% oxygen, and standard laboratory conditions 20% oxygen) and further elevated the mitochondrial activity in NHA cells [37]. Nevertheless, all the drugs were speculated to reverse the malignant phenotype of GSCs isolated from T98G cells since they decreased some of the important GSCs markers. Exposure to agomelatine significantly reduced the expression of CD44, nestin, Sox1 and more prominently, Sox2 (to 0.1%). In a hypoxia-reoxygenation model, the expression levels of CD44, Ki67, Sox1, and Sox2 were found to be related as observed previously in the hypoxia model. Contrary to the hypoxia model, the expression of nestin was significantly elevated compared as depicted by 5% nestin-positive cells. Similar to mirtazapine, when tested under standard laboratory conditions (20% oxygen), GSC marker expressions (CD44, Sox1 and Sox2) were undetected, and Ki67 expression was reduced following treatment with fluoxetine, escitalopram and agomelatine.

Other than inhibiting cell proliferation and inducing glioma cell death, SSRIs have also been reported to hinder the invasion of human glioblastoma by disrupting actin polymerization. Fluvoxamine, which is also a sigma-1 receptor agonist, was reported as the most efficacious drug (compared with dynasore as a positive control) in suppressing actin polymerization (F-actin), with an IC_50_ value of 30 μM [44]. Fluvoxamine (40 μM, 15 min) blocked the formation of lamellipodia in serum-starved U-87MG and U-251MG cells, and suppressed the migration of A172, U87-MG, and U251-MG in a concentration-dependent manner (0, 25 and 50 μM). Although fluvoxamine (10-50 μM) significantly blocked the invasion of U87-MG and human glioma-initiating cells (HGICs), it did not reduce U-87MG and U-251MG cell viability, which suggested its anti-invasion properties are independent of anti-proliferative activity. The anti-invasion activity of fluvoxamine is mediated by the inhibition of PI3K-Akt/mTOR, as observed by reduced FAK phosphorylation, Akt phosphorylation (Thr 308 and Ser 473) and mTOR phosphorylation (Ser 2448 and Ser 2481). Treatment with fluvoxamine (50 mg/kg/day, intraperitoneally) was shown to localize CD133+ cells at tumor sites and reduced the CD31+ cells-positive cells and Ki67 + positive cells in hGICs-xenograft mice, which prolonged their survival from 34 to 41 days.

### Tricyclic anti-depressants uses in glioma studies

Tricyclic antidepressants (TCAs) are a group of anti-depressants that modulate an array of neurotransmitter systems such as histaminic, muscarinic, and alpha-1 receptors. Nowadays, tricyclic anti-depressants have been replaced by a newer generation of drugs that exhibit fewer side-effects, although they are still prescribed at lower doses to treat chronic pain, sleep disorders, panic disorders, and intractable depression. One of the widely used tricyclic anti-depressants in glioma studies is clomipramine (chlorimipramine) which has been reported to possess anti-cancer activity by modulating apoptotic cell death [45], autophagic flux [46], the stemness of cancer stem cells [47], and the reversal of chemotherapeutic drug resistance in a number of cancer models [48].

In earlier studies, clomipramine treatment (2–256 μM, 1 h) significantly reduced the cell viability of astrocytoma primary cultures (grade II) IPDDC-A2, anaplastic astrocytoma (grade III), NP785-96, and glioblastoma multiforme (grade IV) IPTP-98 cells [49]. Furthermore, it suppressed oxygen consumption (0.28, 0.57 and 1.4 mM in 15, 10 and 5 min) down by 95% in IPTP-98 cells. Further treatment with clomipramine (114 μM) significantly blocked mitochondrial complex III activity which reduced MMP, followed by mitochondrial swelling and vacuolation that led to increased caspase-3 activity. In different models, clomipramine (0 – 50 μM for 24 h) reduced C6 and SH-SY5Y cell viability through the induction of apoptotic cell death (when treated with 12 μM) in a manner similar to paroxetine, via the leakage of cyt. *c* and increased activation of caspase-3 [41]. In another apoptotic evaluation study in five different primary glioma cells (SNB-19, DK-MG, UPAB, UPMC, and UPJM), increasing concentrations of clomipramine (0–100 μM, from 1–6 h), induced moderate apoptotic cell death (up to 17 % at 100 μM for 2 h exposure) in SNB-19 cells [50]. In DK-MG cells, exposure of clomipramine for 4 h at 60 μM exhibited a stronger apoptotic effect, up to 51%, and 49% at 100 μM for 6 h. The positive control, staurosporine produced more positive apoptotic cells (71% at 6 h). However, this was an apoptotic mechanism study described by the authors as supporting and corroborating the apoptotic-inducing effects of clomipramine.

A combination treatment study with clomipramine and imatinib (both at 10 μM for 96 h) in C6 cells displayed synergistic inhibition of cell proliferation, viability, and inhibition of DNA synthesis with an enhanced annexin-V-positive cell percentage at 24 h (35.49% increased to 61.95%) and 96 h (80.49% increased to 86.52%) [51]. Furthermore, the addition of clomipramine significantly reduced cAMP levels to 30.40 and 5.19 pmol/ml (at 24 h and 96 h, respectively) as compared with exposure to imatinib alone (61.61 and 37.68 pmol/ml at 24 h and 96 h, respectively). Additionally, the combination treatment also induced synergistic apoptotic cell death in monolayers and spheroid cultures, as well as an increased appearance of vacuoles. In a neuroblastoma model using SH-SY5Y cells, clomipramine (14.23 μM) potentiated the cell viability reduction of vinorelbine (8 μM), and increased the number of apoptotic cells at 24 and 96 h as compared with vinorelbine treatment alone [52]. Addition of clomipramine further decreased cAMP levels after 96 h of treatment, as compared with vinorelbine treatment alone. Although clomipramine alone increased midkine (neurite growth-promoting factor 2 (NEGF2)) levels; however, its addition with vinorelbine led to a further reduction of midkine after 72 h of treatment. Additionally, combination treatment displayed a further reduction of spheroid volume at 24 and 96 h, decreased BrdU-Li-positive cells to 6% as compared with vonorelbine alone (12 %), and was thought to influence the formation of autophagic vacuoles, lipid droplets, membrane blebbing, and mitochondrial damage in cells. In PTEN-null U-87MG human glioma cells, imipramine was demonstrated to induce autophagic cell death rather than cellular apoptosis through the inactivation of the PI3K-Akt/mTOR signaling pathway [53]. The authors first reported that imipramine (60 μM) time-dependently (15–240 min) suppressed the full activation of Akt by blocking its phosphorylation (Ser 473) that was accompanied with mTOR (Ser 2481). The treatment with imipramine (40–60 μM) also increased the number of PI-staining cells in both U87 and C6 cells, whereas its long-term exposure for 7 days reduced colony formation without promoting DNA fragmentation and PARP cleavage. Furthermore, imipramine-treated cells displayed a marked increase of MDC-labeled vesicles, which exemplified the formation of autophagic vacuoles. This observation was supported by the promotion of LC3-I to LC3-II conversion, the formation of autophagosomes via LC3, and suppression of imipramine-induced autophagic cell death following the knockdown of Beclin 1.

Contrary to the mirtazapine, agomelatine, and SSRIs observations both imipramine (10 μM, 24 h) and amitriptyline (10 μM, 24 h) displayed cytotoxicity in T98G cells, although the reduction of cell viability was moderate as compared with Tmz (1 mM, 24 h) exposure. Nevertheless, this could be due to the higher concentration of Tmz used in all of the oxygen models. Moreover, both imipramine and amitriptyline displayed great potential in modulating the plasticity of GSCs by virtue of their ability in downregulating GSCs markers and hence, reversed the malignant phenotype of the GSCs isolated from T98G cells. In an hypoxia model, both imipramine (TCA) and amitriptyline (TCA) downregulated the expression of CD44 (as low as 30.1%, prominently by amitriptyline), nestin (to 2% prominently by imipramine), Sox1 (to 0%, prominently by imipramine) and Sox2 [37]. In contrast, in an average hypoxia model, imipramine and amitriptyline downregulated CD44 expression to 29% and 30%, respectively when compared with untreated cells (38%). Additionally, Ki67 levels were also decreased by imipramine (a 33% reduction) and amitriptyline (a 32% reduction) as compared with 47% expression in the untreated group. In a hypoxia-reoxygenation model, the expression levels of CD44, Ki67, Sox1, and Sox2 were found to be related, as observed previously in the hypoxia model. Contrary to the hypoxia model, the expression of nestin was significantly elevated compared with 5% nestin-positive cells. Under standard laboratory conditions, GSCs marker expressions (CD44, Sox1, and Sox2) were undetectable in any experimental groups, while Ki67 expression was downregulated as compared with control cells.

### Lithium, an old drug with new a perspective against glioma

Numerous empirical data have supported the use of lithium salts as both an antidepressant and a mood stabilizer, making it the gold standard for the treatment of bipolar disorder [54, 55]. Although lithium has been the main front line choice of treatment for bipolar disorder for decades, its prescription rates have been declining due to growing apprehension of its toxicity and side effects that includes nausea, diarrhea, polyuria, polydipsia, tremor, and cognitive impairment [56]. Nonetheless, lithium offers great therapeutic potential in an array of biological processes such as metabolism, neuronal communication, cell proliferation and development, anti-neoplasia, and modulation of neuroinflammation by virtue of its ability to modulate adenylate cyclase (AC), CREB, and GSK-3β [57, 58].

A decade ago, the potential of LiCl (20 mM, exposure for 96 h) was first reported to almost completely mitigate the X12 glioma spheroid cell invasion [59]. In addition, the same treatment paradigm inhibited all of the glioma cells (U87, U87∆EGFR, U251, U373, X12, and X14 cells) migration, and decreased the size of the sphere when compared to control cells treated with NaCl. However, these effects were reversible following the removal of LiCl (at 24 h) but became completely irreversible with exposure of LiCl at 40 mM. Moreover, when the glioma spheroid cells (X12) and U87 cells were immersed in LiCl (20 mM, 48 h), the long protrusions at the front of the glioma cells were retracted and the cells became less elongated. Additionally, it was observed that LiCl only reduced the cell viability up to 20% (at 20 mM, 48 h) in U87 cells but induced cell cycle arrest in the G_2_/M phase, which suggested that LiCl was more potent as an anti-invasion and anti-proliferative agent in glioma cells. The authors further suggested that LiCl exhibited its anti-invasion, anti-migration, and anti-proliferative activities through the inhibition of GSK-3 after the knockdown of either GSK-3α or GSK-3β produced suppression of U373 and X-12 cell migration. The overexpression of Bmi1 promotes cancer invasion and metastasis as observed in various cancer models [60–62], and can suppress the cellular senescence in immortalized mouse embryonic fibroblasts through the repression of the Ink4a/Arf-locus [63, 64]. Bmi1 is also reported to be highly expressed in GBM cell lines and primary brain tumors, particularly in LN319 cells [65]. Moreover, the downregulation of Bmi1 by shRNA knockdown of GSK-3β and LiCl (10 mM) treatment were shown to promote CSCs phenotype differentiation, downregulate Sox2 and nestin expression while upregulating differentiation markers, neuronal marker β-tubulin III, oligodendrocyte-specific marker CNPase and the astrocytic marker GFAP in GBM cell lines. These events were followed by decreased clonogenicity, cell migration, abated neurosphere formation, as well as increased apoptotic events (in combination with Tmz) and cell cycle arrest in the G_2_/M phase. Furthermore, LiCl treatment significantly reduced the CD133+ cell subpopulation up to 60%, whereas in *ex vivo* cells from primary tumor biopsies, the suppression of GSK-3β by LiCl decreased the CD133- cell subpopulation as well as altered the protein levels of stem cell and differentiation markers, particularly downregulating Sox2 expression. These findings further confirmed that LiCl can suppress the promotion of the GBM stem cell pool that is independent of CD133 status.

The somatic mutation of the gene encoding isocitrate dehydrogenase (IDH) is mainly observed in secondary glioblastoma multiforme, since IDH1 and IDH2 mutations can promote the concurrent loss and gain of functions that affect cellular levels of α-ketoglutarate and 2-hydroxyglutarate, respectively [66]. It was also reported that the low level of cellular α-ketoglutarate promotes the stabilization of hypoxia-inducible factor 1-alpha (HIF-1α) leading to tumor resistance and angiogenesis [66, 67]. In view of this, the treatment with LiCl (10 – 50 mM) significantly reduced the proliferation of C6 cells transfected with pEGFP-N1, pEGFP-N1-IDH2, and pEGFP-N1-IDH2^R172G^ [67]. Interestingly, LiCl treatment did not produce any significant difference between C6 cells with IDH2 mutations compared with wild-type IDH2, even though mutations in IDH2 were reported to increase the stability of HIF-1α and tumor resistance. Furthermore, treatment with LiCl further promoted production of proMMP-2 and pro-MMP-9 in all the three transfected C6 groups, inhibited the GSK-3β at Ser 9 phosphorylation, augmented the accumulation of β-catenin in the IDH2^R172G^ nuclei group but reduced β-catenin in the C6 IDH2 mutated group, These findings suggest that LiCl promoted the production MMP-2 and -9 through the inactivation of GSK-3β, which activates the Wnt/β-catenin pathway. Additionally, the stabilization of HIF-1α was reduced, which also led to the suppression of C6 migration when the three cell groups were treated with LiCl, and further indicated that LiCl possesses the ability to inhibit the proliferation and migration potential of C6 glioma cells harboring IDH2 mutation, and hence, shows potential in reducing the invasiveness of glioma cells.

Similar to the observations with clomipramine, the addition of LiCl (200 μM) also significantly decreased SHSY-5Y cell proliferation at 24–96 h [52]. LiCl addition induces a higher population of apoptotic cells as compared with vinorelbine alone and vinorelbine with clomipramine at both 24 h and 96 h. The combination of LiCl and vinorelbine abrogated the cAMP level more effectively than vinorelbine alone, or vinorelbine with clomipramine at 24 and 96 h. Even though LiCl addition did not reduce the midkine level, it reduced the spheroid culture volume and induced both nuclear membrane breakdown and the disappearance of the cellular membranes inside the spheroids culture. In a later study, the combination treatment of LiCl (100 μM) with sorafenib (100 μM) for 72 h was shown to further potentiate the reduction of T98G cell viability and increase the population of apoptotic cell populations in a synergistic manner when compared with the drug treatments alone [68]. Although combination treatment induced the second highest reduction of EGFR, p-STAT-3, p-ERK, p-AKT, p-GSK-3β, NF-κB, and p170 levels as compared with sorafenib treatment alone, the former treatment resulted in the greatest reduction of midkine and multidrug resistance protein 1 (MRP1). Additionally, the combination treatment displayed a higher frequency of ultrastructural damage, with the appearance of apoptotic nuclei and lytic cytoplasm inside the pool of cell remnants as compared with all of the treatment groups. In another study using the antileukemic drug imatinib mesylate (10 μM) by the same group, the addition of LiCl (100 μM) for 72 h produced antagonistic effects, although the anti-proliferation, apoptosis and expression of key proteins involved were found to be significantly modulated when compared with control cells [69]. The same study reported that LiCl treatment alone was more effective in decreasing T98G cell proliferation, EGFR, platelet derived growth factor receptor-alpha (PDGFR-α), MRP-1, aquaporin-4, and cAMP levels as compared with the combination of LiCl and imatinib mesylate. Furthermore, the latter treatment alone more prominently inhibited p-GSK-3β and hence, decreased the ratio of p-GSK-3β/GSK-3β which was followed by LiCl treatment alone. In addition, LiCl alone resulted in a higher frequency of appearances of apoptotic and autophagic vacuoles as compared with the combination treatment. Nonetheless, the combination treatment demonstrated the most profound decrease of p170 levels and further reduced midkine and Bcl-2 levels as compared with LiCl treatment alone. Interestingly, while imatinib mesylate induced G_0_/G_1_ cell cycle arrest, both the combination and LiCl treatment alone produced cell cycle arrest in the G_2_/M phase. These observations suggest that LiCl addition to a chemotherapeutic drug regimen is not necessarily producing additive or synergetic effects, as hypothesized. Nevertheless, the apoptotic and autophagic cell death effects are still significantly induced as compared with the control group. In a more recent study, the combination of cimetidine, LiCl, olanzapine, and valproate, known as the CLOVA cocktail, were shown to suppress GSK-3β by inhibiting pGSS641 in all GBM cells (T98G, U87, U251) more profoundly than treatment with each drug alone [70]. When tested separately, LiCl inhibited T98G and U87 (at 5 mM and 10 mM) and U251 (only at 10 mM) cell proliferation. Additionally, the CLOVA cocktail demonstrated a marked increase in cell proliferation inhibition when compared to Tmz, and this was succeeded by the additive suppression effects of Tmz in GBM cells as compared with Tmz alone. Moreover, the Clova cocktail suppressed GBM cell invasion and proliferation in the nude mouse model by inhibiting pFAKY397 and pFAKY861 phosphorylation and by modifying the subcellular localization of active Rac1 in GBM tumor cells.

### Repurposing the potentiality of valproic acid (VPA) in glioma

#### VPA as anti-proliferative, cell death inducer and anti-angiogenic agent

The ability of valproic acid (VPA) to suppress glioma progression is mainly attributed to its anti-proliferative, anti-angiogenic and apoptotic effects in various glioma models. In an initial study, the exposure of A172, 86HG39, 85HG66, and C6 cells to increasing concentrations of VPA (0.1–1 mM) significantly suppressed cell proliferation in a concentration-dependent manner [71]. Additionally, VPA (1 mM for 7 days) reduced the CD44 antigen in all human glioma cells, while augmenting the expression of CD56. In a later study, VPA exposure (2 mM, 48 h) significantly up-regulated cyclin D3 expression and down-regulated PCNA expression without influencing expression of cyclin D1, cdk1, or the cdk inhibitor kip1/p27 in C6 cells [72]. Further investigation using re-plated synchronized C6 cells demonstrated that VPA induced the acute expression of cyclin D3, which was detectable within 4–6 h in the mid-G_1_ phase. Furthermore, VPA (0.5 mM or 3 mM for 48 h) demonstrated concentration-dependent increases of cyclin D3 expression. However, the observation was reversed (similar to untreated cell at 24 h) when treated cell media was replaced with VPA-free media. Additionally, this observation was supported when VPA (2 mM) exposure for 1 h induced rapid intracellular translocation of cyclin D3 into C6 cell nuclei. The data obtained from this study indicated that modulation of cyclin D3 during G_1_ phase is pertinent to the anti-proliferative effect of that VPA which supported its use as anti-proliferative agent against glioma.

In a recent study, VPA promoted apoptosis in GBM cells by augmenting intracellular ROS levels through the down-regulation of paraoxonase 2 expression [73]. VPA (5–20 mM, 24–72 h) exposure induced significant concentration- and time-dependent reductions in cell viability in the U87, GBM8401, and DBTRG-05MG cells that was coupled with G_2_/M phase arrest and increased sub-G_1_ cell population. These observations were succeeded by the suppression of cell migration and elevation of intracellular ROS levels (at 24–48 h) in all cells. VPA exposure (24 h) significantly downregulated paraoxonase-2, cyclin B1, cdc2, and Bcl-xL expression, while upregulating p21, p27 and Bim expression. Interestingly, the authors observed a negative correlation between paraoxonase-2 and Bim expression where the knockdown of paraoxonase-2 upregulated Bim expression, while the overexpression of paraoxonase-2 reduced intracellular ROS. Moreover, VPA and Tmz (40 μM, 24 h) co-treatment synergistically upregulated Bim but downregulated paraoxonase-2 expression. Additionally, intraperitoneal administration of VPA (400 mg/kg, every two days for 60 days) in the GBM8401 cell xenograft of BALB/c nude mice reduced tumor size (a two-fold reduction) with downregulation of paraoxonase-2 expression that concomitantly elevated intracellular ROS and upregulated Bim expression. Other than mediating ROS-induced apoptosis, VPA also induces autophagic cell death through ROS augmentation in GBM cells [74]. VPA (0.25 to 10 mM for 96 h) significantly reduced T98G, U87MG, and SF295 cell viability in a concentration-dependent manner, while exposure at 1 mM (for 96 h) augmented the apoptotic sub-G_1_ population, reduction of S-phase cell population and induced cell cycle arrest at G_0_/G_1_ phase. The induction of autophagy (VPA, 1 mM for 96 h) was confirmed by the increased presence of autophagic vacuoles and expression of LC3-I, LC3-II, and Beclin-1 in U87MG cells. This indicated that autophagy induction was correlated with the intracellular ROS augmentation via activation of ERK1/2 which was suppressed following pretreatment with N-acetylcysteine and PD98059 in all of the cell lines. Contrarily, co-treatment of VPA with rapamycin, LY294002, and Tmz potentiated autophagy through the PI3K-Akt/mTOR pathway. These observations exemplified that VPA when used alone is capable to promote a different autophagic signaling whereas its combination with pharmacological inhibitors and Tmz potentiated autophagy through the common PI3K-Akt/mTOR signaling. Furthermore, VPA (400 mg/kg/day for 14 days) alone promoted autophagic cell death in U87 xenograft mice by increasing MAP1-LC3 expression in tumor cells while its combination with Tmz (40 mg/kg/day) demonstrated the highest level of autophagic cell death.

Besides inducing apoptosis and autophagy, VPA also exhibits anti-angiogenic activity that mitigates the progression of GBM cells [75]. VPA (0.4–6 mM, 48 h) suppressed GBM cell (U87-MG, U251, A172 and C6 cells) proliferation in a concentration-dependent manner, but with a higher selectivity in TE-1 endothelial cells that first indicated a preference for angiogenic cells. Under both normoxic and hypoxic conditions, VPA concentration-dependently inhibited VEGF secretion in U87-MG and U251 cells. This anti-angiogenic effect was evident VPA (0.5–2 mM) mitigated HUVEC tube formation. A single intraperitoneal VPA (200 mg/kg/day for 28 days) prior to C6 tumor inoculation reduced tumor growth in Wistar rats. This effect was further enhanced when VPA was combined with irinotecan (1 mg/kg/day for 24 days, intraperitoneal). Moreover, VPA alone and combination treatment markedly decreased the cytoplasmic localization of VEGF and vessel densities (total number of factor VIII-positive cells) indicating suppression of angiogenesis. Although p53 mutation status can influence the sensitivity of GBM cells against drug treatment, the exposure of VPA alone (0.75 to 12 mM) for 48–144 h significantly reduced wild-type p53, U87 and mutant p53, LN18 (highest sensitivity) and U251 (lowest sensitivity), with IC_50_ values of 3, 3 and 5.2 mM, respectively (at 72 h) and 2.5, 4, and 1.5 mM, respectively (at 96 h) [76]. Moreover, co-treatment of VPA with etoposide (1.4 to 140 μM) enhanced the cytotoxic effects in all cell lines. Even though VPA alone (1.5 mM, 72 h) induced only a slight increase in G_1_ (U87 cells) and the G_2_/M population (LN18 and U251 cells), co-treatment with etoposide resulted in a significant increase in sub-G_1_ cells and arrestment in G_2_/M phase. These observations highlights the capability of VPA in enhancing chemotherapeutic drugs sensitivity in GBM cells, particularly in mutated p53 GBM cells, possibly through re-activation of p53 signaling. However, such hypothesis merits further investigation to further justify VPA as potential chemotherapeutic drugs sensitizer in GBM cells.

#### VPA as potential epigenetic regulator in glioma

VPA can suppress HDAC activity that leads to nucleosomal histone deacetylation, chromatin condensation, oncogenic silencing, recruitment of tumor suppressor transcription factors, and modulation of cell cycle regulatory proteins acetylation which ultimately influences cellular apoptosis, differentiation and angiogenesis. Interestingly, Das *et al*., (2007) reported that VPA treatment while modulating cell cycle arrestment by upregulating cyclin-dependent kinase inhibitor (CDKI) and p21/WAF1, significantly increased the acetylation of histone H4 (as early as 8 h) for 48 h in GBM cell lines (U87, LN18, U251) [76]. However, both histone H4 acteylation and p21/WAF1 expression declined after 72 h which exemplified a temporal activity of VPA. Additionally, they observed that VPA exposure increased α- and β-isoforms of topoisomerase-II, promoted astrocytic differentiation by upregulating GFAP and caspase-3 activation in all of the cell lines. In another study, VPA treatment (0.5–2 mM for 24–144 h) concentration-dependently inhibited the growth of U87-MG cells together with two other cancer cells, G361 (melanoma) and SK-N-MC (Askin’s tumor) cells [77]. VPA (0.5 and 1 Mm, 24 h) induced apoptosis, modulated histone deacetylase-1 (HDAC-1) expression, suppressed both MMP-2 and MMP-9 and increased TIMP-1 expression which mitigated the cancer cell invasive property.

In another study, VPA was reported to inhibit HDAC and promoted the differentiation of C6 cells into a neuronal-like phenotype by inducing epigenetic changes [78]. VPA (1, 2, 3, 5 & 10 mM) exposure for 24–72 h led to inhibition of C6 cell proliferation with elevation of LDH content in both a concentration- and time-dependent manner. VPA (3 mM) induced histone 4 hyperacetylation followed by inhibition of cell migration and reduction of DNA synthesis as evidenced by reduction in S-phase population (both as compared with FSK as a positive control). Moreover, VPA treatment promoted C6 cells differentiation into a neuronal-like phenotype with neurites and growth cones formation followed by increased βIII-tubulin and decreased GFAP, BDNF, and GDNF protein expression. Based on this, VPA is postulated to induce C6 epigenetic changes that promoted astrocytic-like subpopulation into a neuronal-like phenotype by increasing transgenes expression regulated by neuronal-specific promoters and suppressing transgenes expression controlled by glial-specific promoters. Other than reducing GSC cell viability, VPA (0.25–20 mM, 24–72 h) also induced neuronal-like morphological changes in GSC (GBM2, GBM7, G144, G166, G179, and GliNS2), with cellular astrocytic-like processes [79]. VPA (2 mM) induced the stemness of most GSCs as demonstrated by the increase in CD133 and nestin expression with pro-neuronal differentiation ability, as evidenced by increased βIII-tubulin and GFAP expression. In a follow up study, the exposure of GSCs to VPA at longer periods (14 and 30 days) demonstrated more defined pro-neuronal differentiation with star-shaped and neurite-like processes [80]. Although, G166 and GBM7 cells demonstrated a high percentage of dead cells after the long-term exposure, stemness and differentiation properties of most of the GSCs were maintained as demonstrated with sustained nestin, βIII-tubulin, and GFAP expression with an increase of CD133 and MBP. Additionally, VPA (after 96 h and 30 days) induced oligodendrocyte differentiation activity as shown by increased MBP positive cells and negative expression of other markers in GBM04 cells. In GliNS2 cells, the CD133-, GFAP-, and MBP-positive cells declined, although βIII-tubulin-positive cells were augmented following 14- or 30-days of VPA exposure. In contrast, VPA exposure (30 days) induced the reversal of MGMT promoter methylation to unmethylated in GBM2 and G144 cells. When GBM2 cells were exposed to VPA for 96 h, their sensitivity to Tmz was not improved, which indicates the complex heterogeneity of chemotherapeutic drug resistance in glioma. Apart from inducing the hyperacetylation of H3 protein in SH-SY5Y and SK-N-BE cells, VPA (0.9 and 3 mM) also promoted the reactivation of p53 by inducing its hyperacetylation and nuclear translocation without altering the expression [81]. However, neither the high (3 mM) nor chronic low (0.3 mM for 14 days) administration of VPA induce neuronal morphological changes or differentiation in neuroblastoma cells. Instead, VPA activated the intrinsic apoptotic pathway, induced G_2_ phase arrest, and upregulated the p21/Waf1/Cip1 protein. Additionally, the ability of VPA in regulating p21/Waf1/Cip1 was not detected in the impaired p53 interfered-cells.

Extensive evidence supports the use of epigenetic drugs that inhibit HDAC with the potential reversal of epigenetic alterations that promote tumor cell differentiation by reversing the aberrant changes of chromatin structure to ‘reset’ the cancer cell epigenome. Thus, based on these reported pre-clinical studies, it is noteworthy that the ability of VPA to mimick epigenetic alteration activity further accentuate its therapeutic use as potential therapy by promoting pro-neuronal differentiation that may control the malignancy of glioma. Moreover, the results from Condorelli *et al*., (2008), also support VPA therapeutic use as epigenetic regulator (other than inhibits HDAC activity), by acting as a promiscuous inhibitor of deacetylase enzymes that is capable of modulating the status of functional tumor suppressor protein acetylation. However, this notion still require further investigation since the long term exposure of VPA demonstrated the promoter demethylation of MGMT (in GBM2 and GBM144) which may raise the issue on Tmz chemotherapeutic resistance on different glioma subtypes.

#### VPA as potential adjuvant in chemotherapy and radiation

Although VPA can induce the differentiation of GBM tumors, it main treatment alone is rather cytostasis that only suppress or delay the development of metastases without affecting shrinking or cell death of the GBM tumors. Therefore, a multimodal approach that incorporates the use of chemotherapeutic drugs or irradiation would offer a better therapeutic advantage. Other than inducing the H3 and H4 acetylation index in A172, U373, U138, U87, and SW1783 cells, VPA (1 mM) increased the sensitization towards mitoxantrone, etoposide, and BCNU [82]. VPA (1 mM) resulted in synergistic anti-proliferative effects with significant cell cycle arrest in the S-G_2_/M phases when combined with BCNU. Nevertheless, the combination treatment did not promote cellular apoptosis as compared with BCNU treatment alone. Contrary to this, using different cell lines (LN18 and T98G), VPA treatment (0.5–2 mM, 48 h) followed by taxol and nanotaxol (50 nM, 24 h) synergistically enhanced the cytotoxicity of the drugs [83]. The combination treatment also induced astrocytic differentiation by upregulating GFAP expression (VPA + taxol in T98G cells only and VPA + nanotaxol) and downregulating the inhibitor of differentiation 2 (ID2) in both cells. Additionally, the co-treatment downregulated the expression of VEGF, EGFR, NF-κB, p-Akt, and multidrug resistance (MDR) protein and induced both intrinsic and extrinsic apoptotic pathways. Co-treatment of VPA with Tmz synergistically induced apoptosis in p53-wild type (U87MG cells) and p53-mutant Hs683 models [84]. The apoptotic event was mediated by suppression of nuclear factor-erythroid 2 p45-related factor (Nrf2) nuclear translocation coupled with downregulation of heme oxygenase-1 (HO-1) and γ-glutamylcysteine synthetase (GSH) expression that augmented intracellular ROS production in both cell lines. These findings further corroborated that VPA possesses the ability to enhance apoptotic-inducing effects of Tmz through the interplay of redox regulation rather than the activation of p53 status in GBM cells.

Although, treatment with either Tmz (50 μM) or VPA (2.5 mM) induced autophagic cell death in murine GL261 cells, the combination treatment neither resulted in additive nor synergistic autophagic cell death [85]. Since autophagy can promote tumor antigen presentation by dendritic cells, the co-treatment with Tmz and VPA was postulated to induce adaptive immune responses in both *in vitro* and *in vivo* glioma models. In the same study [84], GL261 cells expressing the membrane-bound form of full-length ovalbumin (GL261mOVA) treated with Tmz and VPA stimulated vigorous induction of proliferation and interferon gamma (IFNγ) production of antigen-specific CD8 T-cells, which suggested that GL261 cells treated with Tmz and VPA were capable of processing and presenting antigens to induce a cytotoxic T-cell immune response. However, none of the Tmz, VPA, or combination pretreated GL261mOVA orthotropic GL261mOVA allograft mice demonstrated improved survival rates (43, 59, and 53.5 days median survival, respectively) as compared with untreated mice (70 days) which indicated that neither Tmz nor VPA promoted immune responses in the allograft mice. Other than conventional chemotherapy, the notion of immunogenic tumor cell death is becoming increasingly pertinent in cancer therapy. It is becomingly known that immunogenic cell death mediates the changes in tumor cell surface composition which promotes the production of mediators that induce trafficking and directing of antigen-presenting cells such as dendritic cells to efficiently present the tumor antigens to T-cells and hence, induces T-cells activation and effector function. By increasing the IFNγ production of antigen-specific CD8 T-cells in *in vitro*, VPA can be postulated to mediate immune-mediated tumor cell killing. However, the incompetency of VPA to promote immunoclearance with lower survival rate (to untreated allograft mice), might dampen this notion, exemplifying the complex tumor immunoregulation that requires further in-depth study.

In a study using GBM cells from 22 patients, VPA (0.8–3.9 mM, 24–48 h) induced sensitivity towards radiation (3 Gy) with cell viability reduction in GS79, GS186, GS224, and GS216 cells. [86]. However, the authors did not further investigate the mechanisms underlying the pre-irradiation sensitization effect of VPA in these models. In Tmz-resistant (T98G) and –sensitive (SF295) cells, the addition of VPA (1 mmol/L, 96 h) increased the sensitivity of the cells towards Tmz and enhanced the irradiation (2 Gy) effect with improved surviving fraction values [87]. This was followed with an augmentation of apoptotic and autophagic cell death associated with cell cycle arrest in the G_2_ phase. However, no further mechanistic work was reported in this study. Likewise, a single treatment with VPA (0.25, 0.5, 1, 2, and 4 mmol/L, 24–72 h) reduced cell viability in a time- and concentration-dependent manner, and induced apoptotic cell death in C6 cells [88]. Additionally, VPA (0.5 mmol/L) significantly enhanced the radiation (2, 4, 6 and 8 Gy) induced cell death and inhibition of clonogenic formation with increased Bax and decreased Bcl-2 mRNA and protein expression. In another study usingVPA and irradiation, the dose-dependent (0.5–2 mM) hyperacetylation of H3 and H4 in SF539 and U251 cells reverted similarly to the control untreated group level following the withdrawal of VPA exposure after 24 h [89]. The same trend of H4 hyperacetylation was observed in U251 xenograft mice treated with VPA (150 mg/kg at 12 h interval for 3 days) where the hyperacetylation rapidly decreased following the withdrawal of VPA. Nevertheless, the exposure of cells to VPA (2 mM in SF539 and 1.5 mM in U251 cells) before and after increasing doses of irradiation (0–8 Gy) enhanced the tumor cell radio-sensitivity, with reduced surviving fraction values. Similarly, VPA administration in combination with irradiation (4 Gy) in U251 xenograft mice prolonged delayed the tumor growth delays as compared to single treatment regimens. Interestingly, VPA alone did not increase γH2AX expression, while irradiation at 10 Gy only sustained γH2AX expression up to 1 h. Nonetheless, the exposure of cells to VPA (before and after irradiation) significantly maintained γH2AX up to 24 h. Since γH2AX is known to assist in the accumulation and retention of DNA damage response proteins, this observation could suggest that the potential therapeutic use of VPA in enhancing irradiation-induced tumor killing via the suppression of double-stranded DNA repair. In a different setting, post-irradiation (2 Gy) exposure of VPA (1.5 mmol/L) was shown to enhance the radio-sensitivity in U251 and SF539 cells [90].

Similar to by Camphausen *et al*., (2005), although VPA alone did not promote acetylation of γH2AX, post-irradiation exposure significantly prolonged irradiation-induced γH2AX and 53BP1 foci dispersal and acetylation of γH2AX up to 24 h. Contrary to this report, pre-treatment with VPA for 24 h in Tmz-resistant T98G cells (2.5 mM) and Tmz-sensitive D384 cells (5 mM) significantly radio-sensitized the γ-radiation-induced cytotoxicity (0–6 Gy), while 24 h post-treatment following irradiation did not influence the cytotoxic activity in both cells [91]. In addition, VPA enhanced Tmz sensitivity in both cell lines while the trimodal treatment regimen (VPA pre-treatment + Tmz (5 μM in D384 and 125 μM in T98G) followed with a single dose of γ-radiation) resulted in the greatest enhancement of cytotoxicity in both Tmz-resistant and –sensitive cells. Increasing VPA exposure (1–16 mM) at clinically achievable concentrations for 96 h led to a significant reduction of cell viability in five different primary GBM cells (MMK1, WK1, SB2, WK2, and LH2) [92]. In a trimodal treatment regime setting, the combination of VPA (1 mM, 24 h), irradiation (5 Gy), and increasing concentrations of Tmz (10–400 μM) displayed the greatest cytotoxicity with an additive effect in three of the primary GBM cells (MMK1, WK1, and SB2 cells). Nevertheless, the dual therapy of VPA and irradiation demonstrated a further reduction of primary GBM cell viability as compared with VPA alone. Moreover, the trimodal treatment regime prolonged overall survival up to 141 days in a pre-treated WK1-transplanted nude mouse model as compared with VPA and irradiation (138 days) and Tmz (115 days). The microarray analysis demonstrated the close clustering between the trimodal treatment regime and the VPA-alone treatment gene expression profile, whereas the Tmz-irradiation regime was closely clustered with untreated cells. The observations from these studies demonstrated that VPA in combination with γ-radiation generally radio-sensitized GBM cells independent of MGMT methylation status. These observations also suggested that VPA addition plays a vital role in the radio-sensitization effects at the gene level in the trimodal treatment regime. In contrast to the Van Nifterik *et al*., (2012) study, pre-incubation with VPA (24 h) and Tmz (40 h) (0.5 mM and 20 μM, respectively), resulted in radio-sensitization effects that were weaker and in some instances, insignificant, in the mutant GBM cell lines p53, T98G, and U87MG [93]. Conversely, the radio-sensitization effects in suppressing cell growth, colony formation, cell death promotion and arrest of the cell cycle in the G_2_/M phase were more pronounced in the wild-type U251MG cells when subjected to fractionated irradiation (single dose of 2 Gy). The contradictory effects of VPA (and in combination with Tmz) radio-sensitization in different p53 status cells in this report could be due to the low clinical concentrations used, since most studies reported cell death-promoting effects of both VPA and Tmz at higher concentrations. Nevertheless, VPA addition was shown to induce immunogenic cell death as it enhanced irradiation-induced HMGB1 and Hsp70 production in p53 mutant and wild-type cells.

One of the biggest concerns of radiation therapy in treating glioma among infants and children is the induction of neurocognitive deficits due to brain injury that can become permanent. Interestingly, pre-incubation with VPA in hippocampal HT22 cells (0.6 mM) and the mouse subgranular zone of the hippocampus (300 mg/kg for 7 days) selectively protected neuronal cells against γ-radiation-induced apoptosis and damage (4 Gy in HT22 and 7 Gy in mice) [94]. Moreover, the radioprotective effect of VPA was accompanied by the upregulation of Bcl-2 and downregulation of Bax proteins in HT22 cells. While doing so, VPA addition prior to γ-radiation induced radio-sensitization as observed by the enhanced G_2_/M cell population (37%) in GL261 glioblastoma cells as compared with γ-radiation (28%). Additionally, VPA (0.6 mM) in combination with increasing doses of γ-radiation (2–8 Gy) significantly reduced the surviving fraction of Daoy, D54, and GL261 cells, while this observation was reversed in HT22 cells. Using dynamic contrast-enhanced magnetic resonance (DCE-MRI), the authors demonstrated enhanced tumor volume reductions in the VPA (300 mg/kg, 5 days) and irradiation (2 Gy, five daily fractions) combination treatment in intracranial orthotopic GL261 mice with 100% 15-day survival rate, as compared with 53% in the irradiation-treated group. Consistent with this result, the same treatment regime delayed tumor growth in heterotopic GL261 (36.5 days) and D54 (24 days) mice as compared with irradiation alone (26.5 days in GL261 and 15.2 days in D54). The findings from this study significantly highlight the potential yet versatile use of VPA as an adjuvant in current GBM therapy that confers radioprotection while enhancing the γ-radiation-induced cell killing selectively in glioma cells. Therefore, based on the reports presented, it is noteworthy for VPA to be considered as an adjuvant therapy to enhance the Tmz and γ-radiation efficacy that may improve the prognosis following the application of Stupp’s regimen.

### Phosphodiesterase-4 inhibitors in glioma

Although phosphodiesterase (PDE) inhibitors became popularly known due to the use of sildenafil for treating erectile dysfunction, in the last decade, PDE inhibitors, particularly PDE-4 inhibitors were discovered to be beneficial in treating psychiatric conditions by increasing cAMP levels. Moreover, inhibition of PDE-4 has been reported to induce anti-cancer activity in a variety of cancer models [95, 96]. Thus, by virtue of their ability to modulate cAMP that further targets an array of molecular cancer targets, PDE-4 inhibitors are suggested to be a potential therapy in treating brain tumors [97]. Treatment with 4-(3-cyclopentyloxy-4-methoxyphenyl)-2-pyrrolidone or rolipram (1–100 μM, for 48 h), a specific phosphodiesterase-4 inhibitor, in the presence of an adenylate cyclase activator, forskolin (to elevate the cAMP levels) was shown to significantly reduce A172 and U87MG cell viability at a lower range of concentrations (10-fold lower) compared with the exposure to non-specific PDE inhibitors, IBMX and theophylline (10–1000 μM, for 48 h) [98]. Additionally, the treatment regime also increased apoptotic cells up to 11.2% (10 and 30 μM rolipram) and increased PDE4B protein expression (0.1 and 10 μM rolipram) in both cells. Rolipram (10 μM) was shown to modulate cAMP levels by augmenting PKA-dependent CREB phosphorylation (Ser 133) and exchange factor directly activated (Epac1)-mediated Ras-proximate-1 (Rap1) activity in A172 cells. Moreover, exposure to the cAMP analogs, dibutyryl-cAMP (dbcAMP) and 8-(4-chloro-phenylthio)-2′-O-methyladenosine-3′,5′-cyclic monophosphate (CPT) induced cell cycle arrest in the G_2_/M phase and reduced A172 cell survival. Co-treatment of dbcAMP or CPT with concentrations of rolipram that induced A172 cell death was further decreased with the addition of H-89, a PKA inhibitor, which further corroborated that rolipram-induced cell death in glioma cells via PKA and Epac1/Rap1 pathway activation. Since overexpression of PDE4A can promote GCSCs cell proliferation through an autocrine mechanism, therefore its application was postulated to suppress glioma cell growth. In view of this, a recent study demonstrated that rolipram enhanced the cytotoxic effect of bevacizumab in human GCSCs (with IC_50_ ~ 6.5 μg/mL) [99]. Moreover, the combination of bevacizumab and rolipram (103 μM) further promoted apoptotic cell death as observed by the activation of caspase-3 through the upregulation of p53, inhibition of Akt phosphorylation (Ser 473), downregulation of VEGF_A,_ and elevation of cAMP levels.

### The potential of pimozide in glioma

Pimozide, discovered in 1963, is an anti-psychotic drug that belongs to the class of diphenylbutylpiperidines that has been used to treat delusional disorders, parasitosis, paranoid personality disorder, Tourette’s syndrome, and resistant tics [100–103]. Pimozide exerts its neurological functions by antagonizing the dopamine D2 receptor subfamily (D2, D3, and D4 receptors), and the 5-HT7 serotonin receptor [104]. The anti-cancer properties of pimozide have been widely reported in various cancer models, including leukemia (by inhibiting STAT-3 and STAT-5), prostate cancer (suppression of STAT-3) [105], pancreatic cancer (antagonist of D2 receptor over-expression) [106], colorectal cancer (blockage of Wnt/β-catenin) [107], and liver cancer (via mitigation of Wnt/β-catenin and STAT-3) [108]. Additionally, pimozide also enhances radiotherapy in breast cancer models and acts as DNA damaging agents by inducing chemo-sensitization and suppressing ubiquitin-specific protease (USP-1) [109, 110]. The initial discovery of the anticancer potential of pimozide in glioma was first reported in its capability to inhibit calmodulin (IC_50_ 6 μM) that correlated with C6 growth inhibition (IC_50_ 10 mM) [111]. Following this, Vilner and Bowen postulated that higher concentrations of pimozide exhibits cytotoxic effects against C6 cells through its potential as a D2 receptor antagonist that has high affinity for σ-receptors. In that study, it was shown that C6 cell viability was decreased with significant morphological changes (rounding of cells) following the exchange of standard culture medium with 100 μM pimozide medium between 18 to 24 h, with a Ki for the σ-receptor of 139 nM [112]. As mentioned, pimozide anti-cancer activity is attributed to its ability to mitigate the activity of USP1/USP-associated factor 1. In a more recent study, the expression of USP1 was reported to be greater in patient-derived glioma cells, especially in GSCs enrichment marker-positive cells (CD133 or CD15) [113]. Since USP1 is beneficial in promoting ID1 and CHEK1 stability that are cardinal in regulating DNA responses and stem cell maintenance, its inhibition is thought to augment DNA damage and glioma cell death. The authors demonstrated that pimozide treatment (5 μM, every three days for two weeks) led to the downregulation of ID1 expression, reduced clonogenic growth, and tumorspheres, decreased glioma stem cell viability, and increased apoptotic cell death as observed by marked upregulation of cleaved caspase-3 and PARP expression. Furthermore, the addition of pimozide (10 mg/kg body weight/day) was shown to induce the radio-sensitivity (2 Gy) that prolonged the survival of GBM xenograft nude mice up to 49 days (two times longer than groups receiving pimozide or radiation treatments alone).

## ANTI-PSYCHOTIC DRUGS AGAINST GLIOMA IN CLINICAL STUDIES

Although increasing evidence has demonstrated the ability of anti-psychotic drugs as potential agents (either as a single or adjuvant therapy) in glioma management, clinical evaluation remains vital in justifying the therapeutic use and confirming prognosis improvement. Over the years, accumulating reports have supported the clinical use of anti-psychotic drugs that improved treatment outcomes with marginal side effects in the current setting of GBM treatment. Moreover, an anti-epileptic drug such as VPA is commonly prescribed to treat glioma patients experiencing seizures. Hence, prospective studies that encompass different statuses of glioma patients are required to decipher the adjuvant role of anti-psychotic drugs and VPA with relevant insights in the current treatment setting.

GBM cells are known to express 5-HT7 receptors, although the exact mechanisms by which serotonin modulates tumor cell growth, proliferation, and stimulation are still undefined. Moreover, anti-psychotic drugs such as SSRIs, TCA, and VPA are commonly co-prescribed during radio-chemotherapy to treat depression, psychosis, and seizures. In a nationwide case-control study in Denmark between January 2000 and December 2012, the long-term use of TCAs among 3767 glioma patients (median age, 60) and 75,340 control population/cancer free (median age, 60) patients showed reduced risk (although statistically not significant) of glioma (OR 0.72, 95% CI: 0.41–1.25) (Pottegård *et al*., 2016). In comparison, the long-term use of SSRIs is not associated with glioma risk (OR 0.93, 95% CI: 0.75–1.16). Although the data collected are based on high-quality nationwide registries, the low number of exposed cases further limits the statistical precision and complicates the interpretation of subgroup analyses in this case-control study. Therefore, this nationwide case-control study requires further validation from association studies and the proper elucidation of the biological rationale to substantiate the efficacy of TCA in reducing the risk of glioma. In a retrospective study involving 160 GBM patients between 1999 and 2008, the intake of SSRIs (citalopram, escitalopram, fluoxetine, fluvoxamine, paroxetine or sertraline) did not induce statistical differences in grade 3 toxicity and no increased toxicity with the use of an SSRI concurrent with treatment of newly-diagnosed GBM when compared with non-SSRIs patients [114]. Although the median survival was 1.05 years, patients who were on SSRIs reported a two-year survival rate of 35% as compared to 17% in patients without SSRIs. Furthermore, they observed that patients taking SSRIs fared better during the year 1 and 2 survival than patients without SSRIs, although it was not statistically significant. The report suggested concomitant use of SSRIs during GBM treatment is safe, and merits further studies in understanding how serotonin acts upon GBM tumor cells. However, this study did not evaluate the efficacy of SSRI use in treating depression among GBM patients. In a recent phase I/II study, the concomitant oral intake of CLOVA cocktail with Tmz (cimetidine, LiCl, olanzapine and VPA) was well-tolerated and safe among seven patients (median age, 66) suffering recurrent GBM in Kanazawa University Hospital (from January 2009 to October 2010) (Furuta *et al*., 2017). Moreover, the concomitant administration of CLOVA cocktail resulted in increased overall survival (11.2 months) compared with the control group treated with TMZ alone (4.3 months). Additionally, the ICC data from the patients’ tissue autopsy revealed decreased levels of pGSS641, nestin, MIB-1 index (pretreatment 32.9% to autopsy 8.0%) and MGMT, which suggests that concomitant intake of CLOVA cocktail enhances Tmz therapy by suppressing tumor invasion via inhibition of GSK-3β activity. These data are similar to the *in vivo* results demonstrated by the same group study which further supported the use of CLOVA cocktail and TMZ as a potential therapy in recurrent GBM patients.

In a retrospective study involving 66 pediatric patients with anaplastic astrocytoma (*n* = 26) and GBM (*n* = 40) aged between 1–19 years old, the addition of VPA to the radio chemotherapy regime did not exacerbate toxicity, with only a single case of pulmonary embolism (in one patient) noted [115]. The findings reported that the best survival was achieved among patients who had complete tumor resection (*p* = 0.0049) while candidates without resection recorded the worst survival rate. Among these, the AA patients demonstrated better overall survival as compared to GBM patients (*p* = 0.0114). The results from this retrospective study suggested VPA use is well-tolerated among AA and GBM pediatric patients and thus provides a scientific basis for more clinical trials in combination with chemotherapeutic drugs or VPA as additions to post-operative radio chemotherapy in glioma patients. In an open-label, phase two study involving 37 GBM patients (18 years and older, median age of 54.3, between July 2006 and April 2013) at the National Cancer Institute (NCI) and Virginia Commonwealth University, the daily intake of VPA (25 mg/kg; 10–15 mg/kg for 1 week, and increased to 25 mg/kg prior to radiation) concurrent with radio-chemotherapy resulted in 29.6 months of median overall survival and 10.5 months of progression-free survival [116]. Additionally, the oral VPA in the chemotherapy regime produced grade 3–4 toxicities, which refer to blood and bone marrow toxicity (32%), neurological toxicity (11%), and metabolic and laboratory toxicity (8%). In a prospective cohort study of pediatric high-grade glioma (HGG; between age 3–18 years; 25 males and 19 females) and diffuse intrinsic pontine glioma (DIPG), VPA (10 mg/kg/day in week 1 and 20 mg/kg/day in week 2 with serum levels of 100–150 mg/L) was given as a maintenance treatment following the completion of eight intensive HITGBM-C chemotherapy cycles (consisting of cisplatin, etoposide, vincristine, and ifosfamide) and radiation [117]. Additionally, VPA was prescribed to patients who had relapsed following intensive chemotherapy before the actual period of VPA intake. Although the median overall survival duration for all patients was 1.33 years, three patients who started VPA intake with progressive tumor status recorded long-term survival durations of 4.5, 4.95, and 5 years. In this study, the efficacy of VPA in improving the prognosis of FGG and DIPG could be improved by combining its use with chemotherapeutic drugs and radiation. Furthermore, the toxicity of VPA was marginal, with no toxicity-related deaths despite the heavy treatment regimen in the patients. A retrospective analysis of 102 GBM patients (median age of 56.3 years) in Taiwan between January 2004 and December 2006 reported survival benefits but not overall patient survival following the administration of VPA with the aim of achieving seizure-free survival [118]. Moreover, tumor sample analysis from a small subset of patients who received VPA demonstrated hyperacetylation of histone, which suggested that VPA may induce HDAC inhibition following the treatment regime that targets seizure control. The report also highlighted the possible benefits of early VPA administration as an adjunct to Tmz, but further work is still required to optimize the dosage schedule that can confer beneficial overall survival among patients.

In a single case-report, a 10-year-old boy with GBM did not respond to partial resection and radio-chemotherapy (54 Gy; vincristine, cisplatin, etoposide, and ifosfamide for 20 weeks; and then topotecan for 10 weeks) regime with seizure complications [119]. Following this, the treatment regime was altered to include VPA with a gradual increase in dosage, with plasma levels ultimately greater than 1 mmol/L (2- to 3-fold above concentrations in children treated for epilepsy) for 10 months was shown to improve the clinical condition, with a reduction in tumor size (complete remission) as evidenced by MRI scanning. When the VPA dosage was reduced due to side-effects, the patient unfortunately suffered relapse 16 months after beginning of VPA treatment. The findings from this report validated the use of VPA as an adjunct therapy in pediatric GBM, and considered for patients with poor response towards radio-chemotherapy. In a rare case of spinal GBM, a 10-month-old infant was treated with electron-beam radiotherapy combined with chemotherapy according to the HIT-GBM-C protocol (cisplatin, etoposide, vincristine, and ifosfamide or every 4 weeks with weekly) [120]. However, the patient’s symptoms recurred less than a month after treatment, with left facial palsy, anisocoria, and hyperhidrosis of the left hemibody. The IHC analysis of the patient’s tumor sample revealed the activation of the Raf-MEK-ERK pathway. As an attempt to inactivate this pathway, the patient was subjected to sorafenib (50 mg twice daily) in combination with VPA (initial dosage of 10 mg/kg/d, and weekly increments of 10 mg/kg) to inhibit HDAC. Prior to a restaging scan, the patient was administered Tmz (160 mg/m2/d) for 5 days and continued with the treatment regime. Interestingly, the treatment regime showed a significant reduction in tumor size, improved symptoms and movements, with continuous improvement until 12 months (treated as an outpatient) without any side-effects. This isolated case study supports the further use and investigation of sorafenib in combination with VPA as a targeted therapy against the MAPK pathway in child GBM. In a Phase 1 Children’s Oncology Consortium trial, the chronic oral administration of VPA (100–150 mcg/mL or 150–200 mcg/mL) in 26 children (age 2 to 21 year-old) with recurrent/refractory glioma was associated with dose-limiting toxicities (somnolence and intra-tumoral hemorrhage), while targeting therapy with concentrations of 75–100 mcg/mL was well-tolerated with mild thrombocytopenia [121]. In this study, the children with high-grade glioma were subjected to VPA intake following radiation, and six cycles of chemotherapy, whereas patients with progressive tumors prior to completing the radio-chemotherapy regime received VPA monotherapy as a recovery therapy. The oral administration of VPA increased peripheral blood mononuclear H3 and H4 histone hyperacetylation in 50% of the pediatric patients, with one patient recording a partial response (VPA troughs 75–100 mcg/mL for 7 months) and another minor response (46% reduction in bi-dimensional measurements; VPA troughs 100–150 mcg/mL for 5 months).

In a retrospective analysis based on the European Organization for Research and Treatment of Cancer (EORTC) and the National Cancer Institute of Canada (NCIC) clinical trial database, the use of VPA was compared with anti-epileptic drugs (AED), enzyme-inducing AED (EIAED, and either one or more; phenytoin, carbamazepine, oxcarbazepine, or phenobarbital) and non-EIAED in 573 patients (age 18–70 years) who received radiotherapy with or without Tmz between 2000 and 2002 [122]. These data first reported that patients administered VPA recorded higher grade (3–4) thrombopenia and leukopenia than patients without AED or patients with AEAED only, but showed no significant differences in anemia. Nevertheless, VPA-group patients demonstrated better survival benefit from the combination with radio-chemotherapy as compared with EIAED only- and non-AED-patients. This retrospective analysis suggests that the inhibition of HDAC activity by VPA most probably plays a role in enhancing radio-chemotherapy efficacy, which correlates with another report of longer survival (14 months vs 11 months) of GBM patients receiving VPA anti-epileptic therapy as an adjunct to the CCNU regime compared with EIAED-patients [123]. A local cohort report involving 236 GBM patients (18 to 78 years with median 62 years) in the United Kingdom also supported the use of radio-chemotherapy in combination NEIAED, particularly, VPA in prolonging the overall survival as compared to AED and EIAED (Guthrie & Eljamel, 2013). Although the AED-group patients recorded longer survivals (11.6 months) compared with EIAED patients, NEIAED patients (mainly VPA) demonstrated significantly longer survival durations of 13.7 months. Another retrospective study among 544 GBM patients (between 18–70 years, median age of 56 years, from 1998–2008) who received other AED and VPA therapies following electron beam radiotherapy and concurrent TMZ regimes revealed the same trend of prolonged overall survival [124]. The initial administration of other AED and VPA was aimed to prevent or reduce seizure complications among patients. VPA intake during radiotherapy prolonged the median overall survival to 16.9 months as compared with AED overall survival of 13.6 months. Moreover, the administration of VPA in combination with Tmz and radiotherapy recorded a longer median overall survival of 23.9 months (15.2 months for AED-group patients). A meta-analysis encompassing five observational studies further corroborated the benefit of VPA using in prolonging the survival of adult GBM patients, with a hazard ratio of 0.74 (95% confidence interval of 0.59–0.94) as compared with other AED [125]. Additionally, the intake of VPA also conferred survival in adult GBM patients with a hazard ratio of 0.66 (95% confidence interval of 0.52–0.84) against the non-AED patients. In a retrospective study among 291 GBM patients who received treatment at the Medical Centre Haaglanden, Netherlands (between July 1999–September 2011), the monotherapeutic use of VPA or levetiracetam in controlling seizures demonstrated freedom from seizures in 77.8% and 69.5% of patients receiving VPA and levetiracetam alone, respectively [126]. When used in combination, the polytherapy regime led to a 60.3% seizure freedom rate, lower than with monotherapy. In addition, patients that received VPA monotherapy in combination with Tmz for a minimum of 3 months displayed prolonged median survival (69 weeks) as compared with the non-VPA monotherapy (61 weeks). A recent retrospective report of 359 glioma patients (grade II – IV) on AED who were treated at the Massachusetts General Hospital with surgery and TMZ (January 1997 - June 2013) demonstrated different treatment outcomes following VPA use among higher grade (IV) and lower grade (II/III) patients [127]. Furthermore, VPA administration in combination with surgical resection and Tmz therapy led to the reduction in death hazard (by 28% in high-grade GBM with improved median overall survival and progression free survival (22 months and 11 months) as compared with other AED patients (14 months and 9 months).

## CONTRADICTORY FINDINGS

The efficacy of anti-psychotic drugs as anti-cancer agents or adjuvants in glioma therapy could be attributed to their therapeutic concentrations. As such, most reports demonstrate their potential therapeutic activities at medium-to-high ranging concentrations either as a monotherapy or in a polytherapy setting. Recent reports that studied the effects of anti-psychotic drugs at a lower range of concentrations might suggest lower concentrations may be worthy of investigation. For instance, although trifluoperazine was reported to dose-dependently (0–30 μM and 1–5 mg/kg/day) promote cellular apoptosis and enhanced autophagic cell death in both *in vitro* and *in vivo* models, one recent finding demonstrates otherwise [128]. The exposure to a low concentration of trifluoperazine (0–10 μM) for 24 h was reported to accelerate cell proliferation and attenuate the induction of Ca^2+^ ions influx which reduced the apoptotic cell death in SWOZ2, SWOZ2-BCNU, and U251 cells. Additionally, the exposure of U251-xenograft nude mice to 2 mg/kg of trifluoperazine further enhanced tumor growth (as observed in Ki67 and PCNA-positive cells), and inhibited apoptosis in the mouse model. It is generally known that anti-psychotic drugs confer the capability to influence the expression of various neurotrophic factors in the brain. Since neurotrophic factor expression can promote and sustain the cancer microenvironment, it is important to investigate the regulation of neurotrophic factors by anti-psychotic drugs at different concentration ranges. In one study the effects of a first-generation neuroleptic drug (haloperidol) and two second-generation neuroleptic drugs (olanzapine and amisulpride) on T98G cells were evaluated, and their influence on neurotrophic factors were measured [129]. Surprisingly, in this study the low range of haloperidol and olanzapine concentrations (5 μM for 72 h) was shown to upregulate BDNF mRNA expression in T98G cells. When further evaluated, olanzapine in particular showed increased BDNF protein expression (by 22% above control values), similar to the positive control cell line PACAP38. The data presented provide a valid concern regarding the low concentration range of AED in glioma therapy in that they seemed to promote protective or tumor-promoting mechanisms in non-neuronal origin cells.

Despite numerous clinical reports that support the use of VPA for prolonging the overall survival of glioma patients, some studies have reported contradictory effects. In an observational analysis among 140 differently graded glioma patients in the Netherlands (between July 2000 until 2005), even though the use of AED (VPA, levetiracetam, carbamazepine, and lamotrigine) led to decreased seizure activity (seizure freedom 59 %), they did not show significantly increased overall survival following radio-chemotherapy [130]. This report was also supported by a prospective report that showed that the administration of VPA and levetiracetam (before or after chemotherapy) was not associated with progression-free survival or overall survival when compared with patients that were not administered AED drugs [131]. The contrary data from these studies suggest that the use of VPA and levetiracetam, in particular, might only be useful in the prevention or control of seizures among glioma patients. Although the use of VPA in radio-chemotherapy was shown to be beneficial in prolonging overall survival and progression-free survival in high grade glioma, lower-grade glioma (grade II/III) showed an inverse correlation with increased risk of tumor progression or death (118 %), reduced overall survival, and progression-free survival (109 months and 44 months) as compared with other AED patients (127 months and 117 months) [127]. The findings from this retrospective study indicated that death hazard or progression was reduced following an increment of every 100 g of VPA intake in high-grade glioma and vice versa in lower-grade glioma patients. Considering the lack of VPA clinical evaluations in different grades of glioma, the findings provided interesting insights regarding the efficacy of VPA administration, as VPA demonstrated a positive correlation outcome in GBM patients but the inverse in lower-grade glioma patients.

## CONCLUSIONS AND FUTURE PERSPECTIVES

Since the current treatment prognosis, particularly in high-grade glioma (Grade III and IV) is hampered with tumor recurrence and chemotherapeutic resistance, curing or even prolonging the overall survival of high-grade glioma patients beyond two years remains an elusive goal. This review describes the use of anti-psychotic drugs in preclinical and clinical studies either as monotherapies or adjuvants in treating human glioma. The long history and clinical experience of anti-psychotic drugs in other cancer models simply justify their potential to be repurposed as cheaper and effective chemotherapeutic agents. Moreover, the use of anti-psychotic drugs as monotherapy agents or adjuvants transcend gender, age, psychological status, and glioma grade among patients. Notwithstanding, the application of anti-psychotic drugs in glioma management still require further and deeper evaluation, particularly in regard to its bioavailability, safety and optimal dosage, tumor multi-resistance and microenvironment as well as potential side-effects. Additionally, the lack of clinical documentation that supports the use of anti-psychotic drugs in inducing cellular differentiation and tumor control in glioma therapy further necessitates its clinical evaluation. In doing so, retrospective studies would also be beneficial in providing information regarding their safety and confirming their influence on survival and response rates in glioma patients. One of the findings in this review accentuated the encouraging and positive outcomes of VPA as an adjunct in improving the symptoms and survival among pediatric glioma patients. In this regard, it would be interesting to determine VPA use in combination with current radio-chemotherapy settings on a larger scale in clinical trials, and hence validate its beneficial therapeutic role.

Most of the collective reports suggest a multimodal therapy approach that includes the use of anti-psychotic drugs as adjuvants to radio-chemotherapy. It is noteworthy that glioma is not a single disease and therefore, a monotherapy regime may not be suitable for every patient. Moreover, polytherapy confers the benefits of targeting multiple treatment issues such as chemoresistance due to multidrug resistant protein, epilepsy or depression onset, chemo- and radio-sensitization and tumor cell killing via pleotropic molecular targets. In doing so, it is essential to determine whether the administration of anti-psychotic drugs may increase Tmz bioavailability during treatment which thereby might explain the radio-chemo-sensitization effects observed in both *in vivo* and clinical studies. Furthermore, the ability of anti-psychotics to target multiple signaling pathways via activation or inactivation of various downstream targets that underlie their anticancer effects in Tmz-resistant and Tmz-sensitive tumors further justifies their use in polytherapy.

Anti-psychotic drugs other than VPA lack clinical evaluation, which might depreciate their preclinical therapeutic efficacy. Hence, extensive retrospective, prospective, observational and randomized clinical studies are required to further validate their preclinical anti-glioma activities. Additionally, clinical evaluations of VPA mainly reported positive impacts on high-grade glioma patients, but confounding observations in limited reports regarding low-grade glioma patients. These contradictory observations of VPA effects in glioma further necessitate wider clinical studies that include different glioma grades to justify its clinical use as an adjuvant in a polytherapy approach. Despite the therapeutic advances offered by anti-psychotic drugs in preclinical and clinical settings, it is noteworthy that most drugs such as LiCl and VPA might possess a narrow therapeutic window and can be toxic at higher doses or chronic administration. Patients reported pausing or stopping the intake of anti-psychotic drugs due to side-effects, and in certain cases patients demonstrated accelerated disease progression or relapse. Additionally, the high- and low-range of concentrations of anti-psychotic drugs also demonstrated contradictory glioma growth activities. Therefore, detailed pharmacokinetic and toxicity analyses are required to determine the ideal concentration that provides optimal therapeutic efficacy with marginal side-effects.

In short, based on various preclinical and clinical studies, anti-psychotic drugs hold substantial therapeutic value not only in the modulation of psychotic symptoms and seizures, but more importantly, as potential anti-neoplastic and adjuvant agents in glioma management. Hence, extensive preclinical and clinical studies would further strengthen the evidence of their therapeutic efficacy and repurpose their use in human glioma management.

## SUPPLEMENTARY MATERIALS




