# Supplementary material for: Emerging therapeutic potential of anti-psychotic drugs in the management of human glioma: A comprehensive review
Source: Oncotarget. 2019 Jun 11;10(39):3952–77. doi: 10.18632/oncotarget.26994 (PMC6570463; doi:10.18632/oncotarget.26994)
Supplement: Supplementary file 1 [file oncotarget-10-3952-s001.pdf]

Emerging therapeutic potential of anti-psychotic drugs in the management of human glioma: A comprehensive review

SUPPLEMENTARY MATERIALS

REFERENCES

1. Mégalizzi V, Decaestecker C, Debeir O, Spiegl-Kreinecker S, Berger W, Lefranc F, Kast RE, Kiss R. Screening of anti-glioma effects induced by sigma-1 receptor ligands: potential new use for old anti-psychiatric medicines. Eur J Cancer. 2009; 45:2893–905. https://doi.org/10.1016/j.ejca.2009.07.011. [PubMed]
2. Sozio P, Fiorito J, Di Giacomo V, Di Stefano A, Marinelli L, Cacciatore I, Cataldi A, Pacella S, Turkez H, Parenti C, Rescifina A, Marrazzo A. Haloperidol metabolite II prodrug: asymmetric synthesis and biological evaluation on rat C6 glioma cells. Eur J Med Chem. 2015; 90:1–9. https://doi.org/10.1016/j.ejmech.2014.11.012. [PubMed]
3. Gil-Ad I, Shtaif B, Levkovitz Y, Dayag M, Zeldich E, Weizman A. Characterization of phenothiazine-induced apoptosis in neuroblastoma and glioma cell lines: clinical relevance and possible application for brain-derived tumors. J Mol Neurosci. 2004; 22:189–98. https://doi.org/10.1385/JMN:22:3:189. [PubMed]
4. Aas AT, Brun A, Pero RW, Salford LG. Chlorpromazine in combination with nitrosourea inhibits experimental glioma growth. Br J Neurosurg. 1994; 8:187–92. https://doi.org/10.3109/02688699409027965. [PubMed]
5. Shin SY, Lee KS, Choi YK, Lim HJ, Lee HG, Lim Y, Lee YH. The antipsychotic agent chlorpromazine induces autophagic cell death by inhibiting the Akt/mTOR pathway in human U-87MG glioma cells. Carcinogenesis. 2013; 34:2080–89. https://doi.org/10.1093/carcin/bgt169. [PubMed]
6. Oliva CR, Zhang W, Langford C, Suto MJ, Griguer CE. Repositioning chlorpromazine for treating chemoresistant glioma through the inhibition of cytochrome c oxidase bearing the COX4-1 regulatory subunit. Oncotarget. 2017; 8:37568–83. https://doi.org/10.18632/oncotarget.17247. [PubMed]
7. Cheng HW, Liang YH, Kuo YL, Chuu CP, Lin CY, Lee MH, Wu AT, Yeh CT, Chen EI, Whang-Peng J, Su CL, Huang CY. Identification of thioridazine, an antipsychotic drug, as an antiglioblastoma and anticancer stem cell agent using public gene expression data. Cell Death Dis. 2015; 6:e1753. https://doi.org/10.1038/cddis.2015.77. [PubMed]
8. Berghauser Pont LM, Balvers RK, Kloezeman JJ, Nowicki MO, van den Bossche W, Kremer A, Wakimoto H, van den Hoogen BG, Leenstra S, Dirven CM, Chiocca EA, Lawler SE, Lamfers ML. *In vitro* screening of clinical drugs identifies sensitizers of oncolytic viral therapy in glioblastoma stem-like cells. Gene Ther. 2015; 22:947–59. https://doi.org/10.1038/gt.2015.72. [PubMed]
9. Otręba M, Buszman E. Perphenazine and prochlorperazine induce concentration-dependent loss in human glioblastoma cells viability. Pharmazie. 2018; 73:19–21. https://doi.org/10.1691/ph.2018.7806. [PubMed]
10. Tzadok S, Beery E, Israeli M, Uziel O, Lahav M, Fenig E, Gil-Ad I, Weizman A, Nordenberg J. *In vitro* novel combinations of psychotropics and anti-cancer modalities in U87 human glioblastoma cells. Int J Oncol. 2010; 37:1043–51. https://doi.org/10.3892/ijo_00000756. [PubMed]
11. Kang S, Hong J, Lee JM, Moon HE, Jeon B, Choi J, Yoon NA, Paek SH, Roh EJ, Lee CJ, Kang SS. Trifluoperazine, a Well-Known Antipsychotic, Inhibits Glioblastoma Invasion by Binding to Calmodulin and Disinhibiting Calcium Release Channel IP3R. Mol Cancer Ther. 2017; 16:217–227. https://doi.org/10.1158/1535-7163.MCT-16-0169-T. [PubMed]
12. Zhang X, Xu R, Zhang C, Xu Y, Han M, Huang B, Chen A, Qiu C, Thorsen F, Prestegarden L, Bjerkvig R, Wang J, Li X. Trifluoperazine, a novel autophagy inhibitor, increases radiosensitivity in glioblastoma by impairing homologous recombination. J Exp Clin Cancer Res. 2017; 36:118. https://doi.org/10.1186/s13046-017-0588-z. [PubMed]
13. Kang S, Lee JM, Jeon B, Elkamhawy A, Paik S, Hong J, Oh SJ, Paek SH, Lee CJ, Hassan AH, Kang SS, Roh EJ. Repositioning of the antipsychotic trifluoperazine: Synthesis, biological evaluation and in silico study of trifluoperazine analogs as anti-glioblastoma agents. Eur J Med Chem. 2018; 151:186–98. https://doi.org/10.1016/j.ejmech.2018.03.055. [PubMed]
14. Shin SY, Choi BH, Ko J, Kim SH, Kim YS, Lee YH. Clozapine, a neuroleptic agent, inhibits Akt by counteracting Ca2+/calmodulin in PTEN-negative U-87MG human glioblastoma cells. Cell Signal. 2006; 18:1876–86. https://doi.org/10.1016/j.cellsig.2006.02.004. [PubMed]
15. Karpel-Massler G, Kast RE, Westhoff MA, Dwucet A, Welscher N, Nonnenmacher L, Hlavac M, Siegelin MD, Wirtz CR, Debatin KM, Halatsch ME. Olanzapine inhibits proliferation, migration and anchorage-independent growth in human glioblastoma cell lines and enhances temozolomide's antiproliferative effect. J Neurooncol. 2015; 122:21–33. https://doi.org/10.1007/s11060-014-1688-7. [PubMed]
16. Bielecka-Wajdman AM, Lesiak M, Ludyga T, Sieroń A, Obuchowicz E. Reversing glioma malignancy: a new look

at the role of antidepressant drugs as adjuvant therapy for glioblastoma multiforme. Cancer Chemother Pharmacol. 2017; 79:1249–56. https://doi.org/10.1007/s00280-017-3329-2. [PubMed]

17. Wang Y, Huang N, Li H, Liu S, Chen X, Yu S, Wu N, Bian XW, Shen HY, Li C, Xiao L. Promoting oligodendroglial-oriented differentiation of glioma stem cell: a repurposing of quetiapine for the treatment of malignant glioma. Oncotarget. 2017; 8:37511–24. https://doi.org/10.18632/oncotarget.16400. [PubMed]

18. Kast RE, Skuli N, Karpel-Massler G, Frosina G, Ryken T, Halatsch ME. Blocking epithelial-to-mesenchymal transition in glioblastoma with a sextet of repurposed drugs: the EIS regimen. Oncotarget. 2017; 8:60727–49. https://doi.org/10.18632/oncotarget.18337. [PubMed]

19. Spanová A, Kovárů H, Lisá V, Lukásová E, Rittich B. Estimation of apoptosis in C6 glioma cells treated with antidepressants. Physiol Res. 1997; 46:161–64. [PubMed]

20. Levkovitz Y, Gil-Ad I, Zeldich E, Dayag M, Weizman A. Differential induction of apoptosis by antidepressants in glioma and neuroblastoma cell lines: evidence for p-c-Jun, cytochrome c, and caspase-3 involvement. J Mol Neurosci. 2005; 27:29–42. https://doi.org/10.1385/JMN:27:1:029. [PubMed]

21. Liu KH, Yang ST, Lin YK, Lin JW, Lee YH, Wang JY, Hu CJ, Lin EY, Chen SM, Then CK, Shen SC. Fluoxetine, an antidepressant, suppresses glioblastoma by evoking AMPAR-mediated calcium-dependent apoptosis. Oncotarget. 2015; 6:5088–101. https://doi.org/10.18632/oncotarget.3243. [PubMed]

22. Hayashi K, Michiue H, Yamada H, Takata K, Nakayama H, Wei FY, Fujimura A, Tazawa H, Asai A, Ogo N, Miyachi H, Nishiki T, Tomizawa K, et al. Fluvoxamine, an anti-depressant, inhibits human glioblastoma invasion by disrupting actin polymerization. Sci Rep. 2016; 6:23372. https://doi.org/10.1038/srep23372. [PubMed]

23. Bilir A, Erguven M, Yazihan N, Aktas E, Oktem G, Sabanci A. Enhancement of vinorelbine-induced cytotoxicity and apoptosis by clomipramine and lithium chloride in human neuroblastoma cancer cell line SH-SY5Y. J Neurooncol. 2010; 100:385–95. https://doi.org/10.1007/s11060-010-0209-6. [PubMed]

24. Nowicki MO, Dmitrieva N, Stein AM, Cutter JL, Godlewski J, Saeki Y, Nita M, Berens ME, Sander LM, Newton HB, Chiocca EA, Lawler S. Lithium inhibits invasion of glioma cells; possible involvement of glycogen synthase kinase-3. Neuro Oncol. 2008; 10:690–99. https://doi.org/10.1215/15228517-2008-041. [PubMed]

25. Korur S, Huber RM, Sivasankaran B, Petrich M, Morin P Jr, Hemmings BA, Merlo A, Lino MM. GSK3β regulates differentiation and growth arrest in glioblastoma. PLoS One. 2009; 4:e7443. https://doi.org/10.1371/journal.pone.0007443. [PubMed]

26. Fu Y, Zheng Y, Chan KG, Liang A, Hu F. Lithium chloride decreases proliferation and migration of C6 glioma cells harboring isocitrate dehydrogenase 2 mutant via GSK-3β. Mol Biol Rep. 2014; 41:3907–13. https://doi.org/10.1007/s11033-014-3258-7. [PubMed]

27. Sabancı PA, Ergüven M, Yazıhan N, Aktaş E, Aras Y, Civelek E, Aydoseli A, Imer M, Gürtekin M, Bilir A. Sorafenib and lithium chloride combination treatment shows promising synergistic effects in human glioblastoma multiforme cells *in vitro* but midkine is not implicated. Neurol Res. 2014; 36:189–97. https://doi.org/10.1179/1743132813Y.0000000283. [PubMed]

28. Aras Y, Erguven M, Aktas E, Yazihan N, Bilir A. Antagonist activity of the antipsychotic drug lithium chloride and the antileukemic drug imatinib mesylate during glioblastoma treatment *in vitro*. Neurol Res. 2016; 38:766–74. https://doi.org/10.1080/01616412.2016.1203096. [PubMed]

29. Furuta T, Sabit H, Dong Y, Miyashita K, Kinoshita M, Uchiyama N, Hayashi Y, Hayashi Y, Minamoto T, Nakada M. Biological basis and clinical study of glycogen synthase kinase- 3β-targeted therapy by drug repositioning for glioblastoma. Oncotarget. 2017; 8:22811–24. https://doi.org/10.18632/oncotarget.15206. [PubMed]

30. Daley E, Wilkie D, Loesch A, Hargreaves IP, Kendall DA, Pilkington GJ, Bates TE. Chlorimipramine: a novel anticancer agent with a mitochondrial target. Biochem Biophys Res Commun. 2005; 328:623–32. https://doi.org/10.1016/j.bbrc.2005.01.028. [PubMed]

31. Parker KA, Pilkington GJ. Apoptosis of human malignant glioma-derived cell cultures treated with clomipramine hydrochloride, as detected by Annexin-V assay. Radiol Oncol. 2006; 40:87–93.

32. Bilir A, Erguven M, Oktem G, Ozdemir A, Uslu A, Aktas E, Bonavida B. Potentiation of cytotoxicity by combination of imatinib and chlorimipramine in glioma. Int J Oncol. 2008; 32:829–39. https://doi.org/10.3892/ijo.32.4.829. [PubMed]

33. Jeon SH, Kim SH, Kim Y, Kim YS, Lim Y, Lee YH, Shin SY. The tricyclic antidepressant imipramine induces autophagic cell death in U-87MG glioma cells. Biochem Biophys Res Commun. 2011; 413:311–17. https://doi.org/10.1016/j.bbrc.2011.08.093. [PubMed]

34. Moon EY, Lee GH, Lee MS, Kim HM, Lee JW. Phosphodiesterase inhibitors control A172 human glioblastoma cell death through cAMP-mediated activation of protein kinase A and Epac1/Rap1 pathways. Life Sci. 2012; 90:373–80. https://doi.org/10.1016/j.lfs.2011.12.010. [PubMed]

35. Ramezani S, Vousooghi N, Kapourchali FR, Hadjighasem M, Hayat P, Amini N, Joghataei MT. Rolipram potentiates bevacizumab-induced cell death in human glioblastoma stem-like cells. Life Sci. 2017; 173:11–19. https://doi.org/10.1016/j.lfs.2017.02.005. [PubMed]

36. Lee GL, Hait WN. Inhibition of growth of C6 astrocytoma cells by inhibitors of calmodulin. Life Sci. 1985; 36:347–54. https://doi.org/10.1016/0024-3205(85)90120-1. [PubMed]

37. Vilner BJ, Bowen WD. Sigma receptor-active neuroleptics are cytotoxic to C6 glioma cells in culture. Eur J Pharmacol. 1993; 244:199–201. https://doi.org/10.1016/0922-4106(93)90029-9. [PubMed]

38. Lee JK, Chang N, Yoon Y, Yang H, Cho H, Kim E, Shin Y, Kang W, Oh YT, Mun GI, Joo KM, Nam DH, Lee J. USP1 targeting impedes GBM growth by inhibiting stem cell maintenance and radioresistance. Neuro Oncol. 2016; 18:37–47. https://doi.org/10.1093/neuonc/nov091. [PubMed]
39. Tseng JH, Chen CY, Chen PC, Hsiao SH, Fan CC, Liang YC, Chen CP. Valproic acid inhibits glioblastoma multiforme cell growth via paraoxonase 2 expression. Oncotarget. 2017; 8:14666–79. https://doi.org/10.18632/oncotarget.14716. [PubMed]
40. Fu J, Shao CJ, Chen FR, Ng HK, Chen ZP. Autophagy induced by valproic acid is associated with oxidative stress in glioma cell lines. Neuro Oncol. 2010; 12:328–40. https://doi.org/10.1093/neuonc/nop005. [PubMed]
41. Condorelli F, Gnemmi I, Vallario A, Genazzani AA, Canonico PL. Inhibitors of histone deacetylase (HDAC) restore the p53 pathway in neuroblastoma cells. Br J Pharmacol. 2008; 153:657–68. https://doi.org/10.1038/sj.bjp.0707608. [PubMed]
42. Chen CH, Chang YJ, Ku MS, Chung KT, Yang JT. Enhancement of temozolomide-induced apoptosis by valproic acid in human glioma cell lines through redox regulation. J Mol Med (Berl). 2011; 89:303–15. https://doi.org/10.1007/s00109-010-0707-1. [PubMed]
43. Knüpfer MM, Hernáiz-Driever P, Poppenborg H, Wolff JE, Cinatl J. Valproic acid inhibits proliferation and changes expression of CD44 and CD56 of malignant glioma cells *in vitro*. Anticancer Res. 1998; 18:3585–89. [PubMed]
44. Bacon CL, Gallagher HC, Haughey JC, Regan CM. Antiproliferative action of valproate is associated with aberrant expression and nuclear translocation of cyclin D3 during the C6 glioma G1 phase. J Neurochem. 2002; 83:12–19. https://doi.org/10.1046/j.1471-4159.2002.01081.x. [PubMed]
45. Das CM, Aguilera D, Vasquez H, Prasad P, Zhang M, Wolff JE, Gopalakrishnan V. Valproic acid induces p21 and topoisomerase-II (α/β) expression and synergistically enhances etoposide cytotoxicity in human glioblastoma cell lines. J Neurooncol. 2007; 85:159–70. https://doi.org/10.1007/s11060-007-9402-7. [PubMed]
46. Proske J, Walter L, Bumes E, Hutterer M, Vollmann-Zwerenz A, Eyüpoglu IY, Savaskan NE, Seliger C, Hau P, Uhl M. Adaptive Immune Response to and Survival Effect of Temozolomide- and Valproic Acid-induced Autophagy in Glioblastoma. Anticancer Res. 2016; 36:899–905. [PubMed]
47. Osuka S, Takano S, Watanabe S, Ishikawa E, Yamamoto T, Matsumura A. Valproic acid inhibits angiogenesis *in vitro* and glioma angiogenesis *in vivo* in the brain. Neurol Med Chir (Tokyo). 2012; 52:186–93. https://doi.org/10.2176/nmc.52.186. [PubMed]
48. Papi A, Ferreri AM, Rocchi P, Guerra F, Orlandi M. Epigenetic modifiers as anticancer drugs: effectiveness of valproic acid in neural crest-derived tumor cells. Anticancer Res. 2010; 30:535–40. [PubMed]
49. Benítez JA, Arregui L, Cabrera G, Segovia J. Valproic acid induces polarization, neuronal-like differentiation of a subpopulation of C6 glioma cells and selectively regulates transgene expression. Neuroscience. 2008; 156:911–20. https://doi.org/10.1016/j.neuroscience.2008.07.065. [PubMed]
50. Riva G, Baronchelli S, Paoletta L, Butta V, Biunno I, Lavitrano M, Dalprà L, Bentivegna A. *In vitro* anticancer drug test: A new method emerges from the model of glioma stem cells. Toxicol Rep. 2014; 1:188–99. https://doi.org/10.1016/j.toxrep.2014.05.005. [PubMed]
51. Riva G, Butta V, Cilibrasi C, Baronchelli S, Redaelli S, Dalprà L, Lavitrano M, Bentivegna A. Epigenetic targeting of glioma stem cells: short-term and long-term treatments with valproic acid modulate DNA methylation and differentiation behavior, but not temozolomide sensitivity. Oncol Rep. 2016; 35:2811–24. https://doi.org/10.3892/or.2016.4665. [PubMed]
52. Ciusani E, Balzarotti M, Calatozzolo C, de Grazia U, Boiardi A, Salmaggi A, Croci D. Valproic acid increases the *in vitro* effects of nitrosureas on human glioma cell lines. Oncol Res. 2007; 16:453–63. https://doi.org/10.3727/096504007783338340. [PubMed]
53. Roy Choudhury S, Karmakar S, Banik NL, Ray SK. Valproic acid induced differentiation and potentiated efficacy of taxol and nanotaxol for controlling growth of human glioblastoma LN18 and T98G cells. Neurochem Res. 2011; 36:2292–305. https://doi.org/10.1007/s11064-011-0554-7. [PubMed]
54. Hosein AN, Lim YC, Day B, Stringer B, Rose S, Head R, Cosgrove L, Sminia P, Fay M, Martin JH. The effect of valproic acid in combination with irradiation and temozolomide on primary human glioblastoma cells. J Neurooncol. 2015; 122:263–71. https://doi.org/10.1007/s11060-014-1713-x. [PubMed]
55. Pont LM, Naipal K, Kloezeman JJ, Venkatesan S, van den Bent M, van Gent DC, Dirven CM, Kanaar R, Lamfers ML, Leenstra S. DNA damage response and anti-apoptotic proteins predict radiosensitization efficacy of HDAC inhibitors SAHA and LBH589 in patient-derived glioblastoma cells. Cancer Lett. 2015; 356:525–35. https://doi.org/10.1016/j.canlet.2014.09.049. [PubMed]
56. Shao CJ, Wu MW, Chen FR, Li C, Xia YF, Chen ZP. Histone deacetylase inhibitor, 2-propylpentanoic acid, increases the chemosensitivity and radiosensitivity of human glioma cell lines *in vitro*. Chin Med J (Engl). 2012; 125:4338–43. [PubMed]
57. Zhou Y, Xu Y, Wang H, Niu J, Hou H, Jiang Y. Histone deacetylase inhibitor, valproic acid, radiosensitizes the C6 glioma cell line *in vitro*. Oncol Lett. 2014; 7:203–08. https://doi.org/10.3892/ol.2013.1666. [PubMed]
58. Camphausen K, Cerna D, Scott T, Sproull M, Burgan WE, Cerra MA, Fine H, Tofilon PJ. Enhancement of *in vitro* and *in vivo* tumor cell radiosensitivity by valproic acid. Int J Cancer. 2005; 114:380–86. https://doi.org/10.1002/ijc.20774. [PubMed]

59. Chinnaiyan P, Cerna D, Burgan WE, Beam K, Williams ES, Camphausen K, Tofilon PJ. Postradiation sensitization of the histone deacetylase inhibitor valproic acid. Clin Cancer Res. 2008; 14:5410–15. https://doi.org/10.1158/1078-0432.CCR-08-0643. [PubMed]

60. Van Nifterik KA, Van den Berg J, Slotman BJ, Lafleur MV, Sminia P, Stalpers LJ. Valproic acid sensitizes human glioma cells for temozolomide and γ-radiation. J Neurooncol. 2012; 107:61–67. https://doi.org/10.1007/s11060-011-0725-z. [PubMed]

61. Duenas-Gonzalez A, Candelaria M, Perez-Plascencia C, Perez-Cardenas E, de la Cruz-Hernandez E, Herrera LA. Valproic acid as epigenetic cancer drug: preclinical, clinical and transcriptional effects on solid tumors. Cancer Treat Rev. 2008; 34:206–22. https://doi.org/10.1016/j.ctrv.2007.11.003. [PubMed]

62. Berendsen S, Broekman M, Seute T, Snijders T, van Es C, de Vos F, Regli L, Robe P. Valproic acid for the treatment of malignant gliomas: review of the preclinical rationale and published clinical results. Expert Opin Investig Drugs. 2012; 21:1391–415. https://doi.org/10.1517/13543784.2012.694425. [PubMed]

63. Wen PY, Schiff D. Valproic acid as the AED of choice for patients with glioblastoma? The jury is out. Neurology. 2011; 77:1114–5. https://doi.org/10.1212/WNL.0b013e31822f0325. [PubMed]

64. Weller M. Are we ready for a randomized trial of valproic acid in newly diagnosed glioblastoma? Neuro Oncol. 2013; 15:809–10. https://doi.org/10.1093/neuonc/not095. [PubMed]

65. Ochiai S, Nomoto Y, Yamashita Y, Watanabe Y, Toyomasu Y, Kawamura T, Takada A, Ii N, Kobayashi S, Sakuma H. Roles of valproic acid in improving radiation therapy for glioblastoma: a review of literature focusing on clinical evidence. Asian Pac J Cancer Prev. 2016; 17:463–66. https://doi.org/10.7314/APJCP.2016.17.2.463. [PubMed]

66. Rudà R, Pellerino A, Soffietti R. Does valproic acid affect tumor growth and improve survival in glioblastomas? CNS Oncol. 2016; 5:51–3. https://doi.org/10.2217/cns-2016-0004. [PubMed]

67. Tan SK, Jermakowicz A, Mookhtiar AK, Nemeroff CB, Schürer SC, Ayad NG. Drug repositioning in glioblastoma: A pathway perspective. Front Pharmacol. 2018; 9:218. https://doi.org/10.3389/fphar.2018.00218. [PubMed]

Supplementary Table 1: The summary of pre-clinical use of anti-psychotic drugs in glioma models

See Supplementary Table 1
