# Supplementary material for: Emerging therapeutic potential of anti-psychotic drugs in the management of human glioma: A comprehensive review
Source: Oncotarget. 2019 Jun 11;10(39):3952–77. doi: 10.18632/oncotarget.26994 (PMC6570463; doi:10.18632/oncotarget.26994)
Supplement: Supplementary file 2 [file oncotarget-10-3952-s002.docx]

**Supplementary Table 1: The summary of preclinical use of anti-psychotic drugs in glioma models**

| Drug | Mechanisms of action in glioma |
| --- | --- |
| Haloperidol | Suppression of growth and cell division in U373 and T98G [1]. |
| (R)-(+)-MRJF4, (S)-(-)-MRJF4 | Promoted apoptosis, cell cycle arrest (S-phase), H3 acetylation and suppressed cell migration in C6 cells [2]. |
| Chlorpromazine | - Reduced C6 cell viability and IC_50_ value of 18.8 µM [3].  - 10 mg/kg body weight, in combination with BCNU inhibited tumor growth in rats injected with RG2 cells [4]. - Inhibited U87MG cell proliferation and promoted autophagic cell death by inhibiting PI3K-Akt/mTOR pathway [5]. - Suppressed U251-derived TMZ-resistant cell proliferation, inhibited CcO complex IV mitochondrial activity and G_1_ phase arrestment. Prolonged overall survival in xenograft UTMZ mice [6]. |
| Thioridazine | - Reduced C6 cell viability (IC_50_, 13.7 µM) and DNA fragmentation. Induction of apoptosis and activation of caspase-3 in SH-SY5Y cells [3]. - Reduced GBM8401 and U87MG cell viability while activating autophagic cell death, apoptosis and ER stress pathway in GBM8401 cells. Suppression tumorigenesis via autophagic cell death in U87-xenograft nude mice [7]. |
| Fluphenazine | - Reduced C6 cell viability (IC_50_,19 µM) and DNA fragmentation [3]. - Sensitized apoptosis and necrosis induction of OV Delta24-RGD in glioblastoma stem-like cells (GSCs) [8]. |
| Perphenazine, Prochlorperazine | - Reduced C6 cell viability (IC_50_, 19 µM) and DNA fragmentation [3]. - Reduced U87-MG cell viability (EC_50_ values 0.98 μM and 0.97 μM) [9]. - Combination with irradiation and Tmz reduced U87 cell viability. Additive effect in activity with while synergistic effect in combination with imatinib [10]. |
| Trifluoperazine | - Reduced U87MG cell viability, motility, invasion and elevated Ca^2+^ ions release. 5 mg/kg/day, inhibited the tumor growth in U87MG-xenograft nude mice [11]. - Reduced radio-sensitivity resistance in GBM models by inhibiting autophagy [12]. - Reduced U251, U87 and P3 cell viability, activated caspase-3/7 and inhibited autophagy by increasing LC3B-II and p62. Enhanced radiation-induced DSBs by prolonging the γ-H2AX signal and downregulating Rad51, BRCA1 and BRCA2 in U251 and U87 cells. Combination with radiation (5 Gy) inhibited tumor proliferation, improved survival and elevated γ-H2AX signal in U251 and P3 nude mice [4]. |
| Trifluoperazine analogs | Analog 3dc reduced tumor size (by 88%) and prolonged survival time (increased by 6 days) in xenograft U87MG nude mice [13]. |
| Clozapine | Inactivation of Akt that interrupted VGCC and CaM, suppression of U-87MG proliferation, inhibition of cyclin D1 and cell cycle arrestment in G_0_/G_1_ phase [14]. |
| Olanzapine | - Promoted Tmz anti-proliferative activity in U87MG, A172, and GSLCs (IC_50_ values from 25 to 79.9 µM) [15]. - Suppressed cell migration and induced cytostatic effect in A172 cells [15]. - Induction of apoptosis and necrosis in A172 cells while apoptosis in U87MG cells via inactivation of AMPK and WNT/β-catenin [15]. |
| Mirtazapine | - Downregulated Sox1 and Sox2 expression in hypoxic T98G cells [16]. - Undetectable expression of CD44, Sox1, and Sox2 and reduced expression of Ki67 in 20% oxygen model [16]. |
| Quetiapine | - Increasing concentration (0-100 μM) reduced GSCs cell viability and arrested cell cycle at G_2_/M at 25 μM [17]. - Increased MBP-positive cells, ODLCs lineage marker MBP and Olig1 expression while reduced GFAP-positive cells and GSCs marker SOX2 [17]. - Inhibited tumor growth and PCNA-positive cells in heterotopic GSC-xenografted nude mice and orthotropic xenografted C57 mice. Enhancement of tumor growth suppression in combination with Tmz [17]. - Re-differentiation of GSCs into ODLCs *in vivo* and inhibited EMT transition [18]. |
| Fluoxetine | - Combination with irradiation (0-8 Gy) reduced U87 cell viability and combination with imatinib elevated the DNA fragmentation, inhibited pAkt and pMAPK expression [9]. - Undetected expression of CD44, Sox1 and Sox2 in 20% oxygen T98G cells [16]. - Induced apoptosis and DNA fragmentation in C6 cells [19]. - Reduction in cell viability, DNA fragmentation and apoptosis in C6 and SH-SY5Y cells [20]. - Decreased cell viability and promoted apoptosis in C6, U87, GBM8401 and Hs683 cells via Ca^2+^ influx and AMPAR receptor. Oral administration (10 mg/kg/day) reduced tumor growth (day 6) which became undetectable (day 12) in U87-xenograft nude mice [21]. |
| Escitalopram | Undetected expression of CD44, Sox1 and Sox2 in 20% oxygen T98G cells [16]. |
| Paroxetine | - Reduction in cell viability, DNA fragmentation and apoptosis in C6 and SH-SY5Y cells [20]. - Promotion of caspase-3 activity, Cyt. *c* release and activation of phospho-c-Jun in C6 cells [20]. |
| Sertraline | - Combination with irradiation (0-8 Gy) reduced U87 cell viability [9]. - Combination with increasing doses of Tmz and imatinib resulted in additive and synergistic reduction of U87 cell content [9]. - Combination with imatinib inhibited pAkt [9]. |
| Fluvoxamine | - Suppressed lamellipodia formation, cell migration and invasion in serum-starved U-87MG and U-251MG cells by inhibiting PI3K-Akt/mTOR [22]. - 50 mg/kg/day (IP) localized CD133+ cells at tumor sites, reduced the CD31+ and Ki67+ positive cells and prolonged survival in hGICs-xenograft mice [22]. |
| Lithium/Lithium Chloride (LiCl) | - Decreased SHSY-5Y cell proliferation (24 – 96 h) and combination with vinorelbine attenuated cAMP level. Reduced spheroid culture volume with nuclear membrane breakdown and cellular membranes disappearances [23]. - Exposure of 20 mM for 96 h almost completely mitigated X12 glioma spheroid cell invasion [24]. Promotion of anti-invasion, anti-migration, and anti-proliferative activities through the inhibition of GSK-3 in U373 and X-12 cell migration. - Same treatment inhibited U87, U87∆EGFR, U251, U373, X12, and X14 cell migration and decreased sphere size but reversible following the removal of LiCl (at 24 h) but became irreversible with at 40 mM [24]. - Promoted CSCs phenotype differentiation by downregulating Bmi1, Sox2 and nestin and upregulating differentiation markers, neuronal marker β-tubulin III, oligodendrocyte-specific marker CNPase and GFAP in GBM cell lines [25]. - Reduced clonogenicity, cell migration, neurosphere formation with promotion of apoptosis and G_2_/M phase arrestment. Decreased CD133+ cell subpopulation, CD133- cell subpopulation and downregulated Sox2 expression in primary tumor biopsies [25]. - Reduced proliferation of transfected IDH2 mutated C6 cells (pEGFP-N1, pEGFP-N1-IDH2, and pEGFP-N1-IDH2^R172G^) and suppressed migration and invasion by promoting pro-MMP-2 and -9 via activation the Wnt/β-catenin pathway and destabilization of HIF-1α [26]. - Combination treatment (100 µM) with sorafenib (100 µM) potentiated reduction of cell viability and apoptosis in T98G cells by suppressing EGFR, p-STAT-3, p-ERK, p-AKT, p-GSK-3β, NF-κB, p170 levels midkine and MRP1 expression [27]. Combination treatment induced higher frequency of ultrastructural damage in T98G cells. - LiCl alone effectively decreased T98G cell proliferation, EGFR, PDGFR-α, MRP-1, aquaporin-4, and cAMP levels with higher frequency apoptotic and autophagic vacuoles with G_2_/M phase arrestement as compared combination of LiCl and imatinib mesylate (produced antagonistic effects) [28]. However, combination with imatinib mesylate decreased p170 levels, midkine and Bcl-2 levels more efficiently than LiCl alone. - CLOVA cocktail (cimetidine, LiCl, olanzapine, and valproate) suppressed GSK-3β by inhibiting pGSS641 in all GBM cells (T98G, U87, U251) more profoundly than treatment with each drug alone [29]. CLOVA enhanced suppression of GBM proliferation and invasion by inhibiting pFAKY397 and pFAKY861 and modifying localization of active Rac1. - CLOVA induced additive suppression effects of Tmz in GBM cells as compared with Tmz alone [29]. |
| Clomipramine | - Reduced of cell viability and activated apoptotic cell death in C6 and SH-SY5Y cells [19]. - Potentiated vinorelbine reduced cell viability, cAMP level and induced apoptosis and autophagy in SH-SY5Y cells [23]. - Reduction of astrocytoma primary cultures (grade II) IPDDC-A2, anaplastic astrocytoma (grade III), NP785-96, and GBM IPTP-98 cell viability. Suppression of O_2_ consumption and MMP that activated caspase-3 activity in IPTP-98 cells [30]. - Moderate apoptotic cell death in SNB-19 cells and strong apoptosis effect in DK-MG cells [31]. - Combination with imatinib synergistically C6 proliferation and induced apoptotic cell death while reducing cAMP levels [32]. |
| Imipramine | - Moderate cytotoxicity and reversed malignant phenotype of the GSCs isolated from T98G cells. Downregulated CD44, Ki67, Sox1, Sox2 and nestin, Sox1 and Sox2 expression [16]. - Induction of autophagic cell death via inactivation of the PI3K-Akt/mTOR in PTEN-null U-87MG cells [33]. |
| Amitriptyline | Modulating the plasticity of GSCs isolated from T98G cells by downregulating CD44, Ki67, Sox1, Sox2 and nestin, Sox1 and Sox2 expression [16]. |
| Rolipram | - Decreased cell viability (at low concentration), induced apoptosis and downregulated PDE4B protein expression in A172 and U87MG in the presence of forskolin [34]. Arrest A172 cell cycle arrest in G_2_/M phase and promoted apoptosis via PKA and Epac1/Rap1 pathway activation. - Enhanced bevacizumab cytotoxicity and promotion of apoptosis via activation of p53 and inhibition of Akt and VEGF_A_ [35]. |
| Pimozide | - Inhibited calmodulin (IC_50_ 6 μM) and C6 cell growth inhibition (IC_50_ 10 mM) [36]. - Conditioned culture medium (100 μM, 18 to 24 h) reduced cell viability and altered morphology of C6 cells [37]. - Downregulated ID1 expression, reduced clonogenic growth and tumorspheres, decreased patient-derived GSC viability, and induced apoptosis. 10 mg/kg body weight/day enhanced the radio-sensitivity (2 Gy) and prolonged the survival in xenograft nude mice [38]. |
| Valproic Acid | - Induction of apoptosis, augmentation of intracellular ROS by downregulation of paraoxonase-2, Nrf2, HO-1 and GSH expression [39–42]. - Anti-proliferative effect with cell cycle arrestment (G_1_, G_2_/M, G_0_/G_1_) by increasing Cyclin D3, p21, p27 and decreasing cyclin B1 and cdc2 [39, 41, 43–45]. - Autophagic cell death induction through the upregulation of LC3-I, LC3-II, Beclin, MBP-LC3 and PI3K-Akt/mTOR and ERK signaling [40, 46]. - Suppression of VEGF, EGFR, MMP-2, MMP-9 that promoted anti-angiogenesis activity and inhibition of cell migration and invasion [47, 48]. - Epigenetic regulation by inhibiting HDAC activity (hyperacetylation of H3, H4) and reactivation of p53 signaling [41, 45, 48, 49]. - Induction of pro-neuronal and oligodendrocyte differentiation by reducing GFAP, BDNF, GDNF and increasing βIII-tubulin, nestin, CD133 and MBP-positive cells [50–52]. - Modulation of immunogenic cell death by stimulating production of IFNγ antigen-specific CD8 T cells and enhanced irradiation induced HMGB1 and HsP70 [42]. - Increase sensitization towards Tmz and chemotherapeutic drugs [53–56]. - Prolonged irradiation induced γH2AX acetylation signal [54–60]. - The preclinical and clinical review of VPA on glioma and other solid tumors [61]. - Review of preclinical and clinical use of VPA in treating malignant gliomas [62]. - Editorial review on the use of VPA as antiepileptic drug for glioblastoma patients [63]. - Editorial review of VPA to be used in randomized trial on newly diagnosed glioblastoma [64]. - Clinical review of VPA in improving radiation therapy for glioblastoma [65]. - Editorial review of VPA use in against glioblastoma in preclinical and improving survival clinical studies [66]. - Review of VPA, other anti-psychotic and non-psychiatric drugs in preclinical and clinical glioblastoma [67].   *Due to the extensive literature review of VPA in the pre-clinical and clinical experimental use, VPA in this section is summarized based on previously published reviews. However, any relevant reports that are not described in this list of reviews are discussed and described in details in the main text. |
